# iSuRe-Cre is a genetic tool to reliably induce and report Cre-dependent genetic modifications

**DOI:** 10.1038/s41467-019-10239-4

**Published:** 2019-05-22

**Authors:** Macarena Fernández-Chacón, Verónica Casquero-García, Wen Luo, Federica Francesca Lunella, Susana Ferreira Rocha, Sergio Del Olmo-Cabrera, Rui Benedito

**Affiliations:** Molecular Genetics of Angiogenesis Group, Centro Nacional de Investigaciones Cardiovasculares (CNIC), Madrid, E28029 Spain

**Keywords:** Cell biology, Developmental biology, Genetics

## Abstract

Most biomedical research aimed at understanding gene function uses the Cre-Lox system, which consists of the Cre recombinase-dependent deletion of genes containing LoxP sites. This system enables conditional genetic modifications because the expression and activity of the recombinase Cre/CreERT2 can be regulated in space by tissue-specific promoters and in time by the ligand tamoxifen. Since the precise Cre-Lox recombination event is invisible, methods were developed to report Cre activity and are widely used. However, numerous studies have shown that expression of a given Cre activity reporter cannot be assumed to indicate deletion of other LoxP-flanked genes of interest. Here, we report the generation of an inducible dual reporter-Cre mouse allele, iSuRe-Cre. By significantly increasing Cre activity in reporter-expressing cells, iSuRe-Cre provides certainty that these cells have completely recombined floxed alleles. This genetic tool increases the ease, efficiency, and reliability of conditional mutagenesis and gene function analysis.

## Abstract (web summary)

The Cre-Lox system allows high spatiotemporal control of genetic modifications. Here the authors present iSuRe-Cre that significantly increases Cre activity in reporter expressing cells, which ultimately increases the efficiency and reliability of Cre-dependent reporter and gene function analysis.

## Introduction

The *Cre-Lox* system enables recombination of a pair of short (34 bp) DNA sequences called *Lox* sequences by the recombinase encoded by the bacteriophage P1 gene *Cre*. This technology has revolutionized biomedical science because it can be used to carry out genetic deletions, inversions, and translocations at specific engineered sites in the genome. Today, most mouse genes have been flanked by *LoxP* sites (floxed), and mouse lines are available expressing constitutively active Cre or tamoxifen-inducible CreERT2 in almost any cell type^1–3^. The availability of these genetic resources enables the precise conditional deletion of almost any mouse gene in any cell type and at a defined time-point, which are crucial requirements for understanding the function of genes during organ development and disease. However, numerous studies demonstrate the need for caution in the use of this powerful technology^4–6^. Many transgenic mouse strains expressing Cre or CreERT2, when interbred with other strains containing floxed alleles, produce highly variable expression patterns and phenotypes that are not apparent when the mice were first created^1,7^. This is partly due to the inconsistent nature or epigenetic silencing of the promoters used in Cre or CreERT2 transgenic mouse lines^8,9^. Two other important issues with Cre-Lox technology are the variable recombination or gene-deletion efficiency and the methods used to detect it. The efficiency of Cre recombination depends on the location of the *LoxP* sites in the genome and the distance separating them^10^. Thus, one should expect a different recombination efficiency for every floxed allele, even when using the same Cre- or CreERT2-expressing mouse line. It is therefore critical to have a reliable method to confirm that a given gene is properly deleted, and only in the desired cell type. Fast PCR based methods are frequently used to confirm genetic deletions in whole tissues or groups of isolated cells, but these methods are insufficient because they do not provide in situ and single-cell resolution. Moreover, these methods only indicate the average gene-deletion efficiency, and cannot quantify the heterogeneity in genetic deletion efficiency among cells. The safest method for confirming inducible and specific gene deletion is to co-immunostain for the encoded protein and a tissue or cell marker. However, for many proteins there are no antibodies able to distinguish between the morphology of cells with and without protein expression in the tissue. Another issue is that gene transcription and protein stability oscillate in a cell, and thus a cell with no detectable expression of a given protein at a given moment may still be wild-type for the coding gene.

To overcome some of these technical problems with the Cre-Lox system, scientists have generated reporters of Cre/LoxP recombination, and these have become widespread and essential genetic tools in any laboratory performing genetic studies. These reporters are usually alleles targeted to the ubiquitous mouse ROSA26 locus^11,12^ and are activated or expressed only after the cell expresses Cre or has induced CreERT2 activity. However, since there is no genetic linkage between the reporter allele and other floxed alleles in the cell, it is unsafe to assume correlation between recombination of the reporter and the target allele. Indeed, several studies have highlighted the unreliability of Cre/LoxP recombination reporters, reporting discrepancies between multiple reporter alleles and target allele recombination^4,6^.

Being able to rely on results arising from the use of Cre/LoxP technology is essential for the progress of biomedical science. Here, we report the generation and utility of an inducible dual reporter-Cre mouse line, called *iSuRe-Cre*. This mouse line is compatible with all existing Cre/CreERT2 and floxed mouse lines and significantly boosts Cre activity in reporter-expressing cells, ultimately increasing the efficiency and reliability of Cre-dependent reporter and gene function analysis.

## Results

### Design and testing of *iSuRe-Cre* constructs

**Fig. 1:**
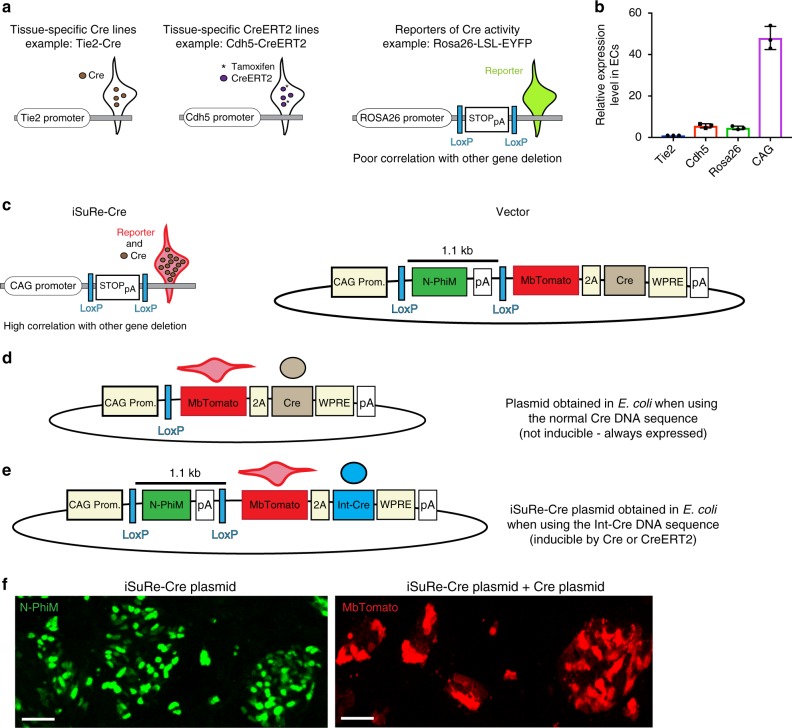
Design and pre-validation of *iSuRe-Cre* plasmids. **a**, **b** Cre or CreERT2 expression from transgenes carrying gene-specific promoters (i.e. *Tie2* or *Cdh5*) is variable and relatively weak (endothelial RNA samples, *n* = 3 animals). In addition, in the case of CreERT2-expressing alleles, tamoxifen needs to be provided to induce activity, of only a fraction of available CreERT2 proteins, and in a limited time window. Due to the variability in Cre expression and induced activity, commonly used Cre activity ROSA26 reporter alleles do not guarantee full recombination of other floxed alleles in the same cell. **c** The new iSuRe-Cre genetic tool enables stronger (CAG promoter driven) and constitutive co-expression of a reporter (MbTomato) and Cre, which is expected to increase the correlation between reporter expression and gene deletion. LoxP, short DNA sequences recognized and recombined by Cre; N-PhiM, nuclear-localized and mutated PhiYFP; pA, Sv40 polyadenylation sequence; 2 A, self-cleaved viral 2 A peptide to guarantee equimolar expression but separate localization of the reporter and Cre. WPRE, Woodchuck hepatitis virus posttranscriptional regulatory element to enhance expression. **d** Vectors containing the normal Cre sequence always had deletion of the N-PhiM-pA cassette in *E. coli*, rendering them non-inducible. **e** The use of an intron-containing Cre sequence (Int-Cre) disrupts Cre expression and function in *E. Coli*, allowing the generation of a Cre-inducible, Cre-expressing vector (iSuRe-Cre). **f** Confocal micrographs showing that cells transfected with iSuRe-Cre plasmids express the N-PhiM protein in the nuclei. If cells are co-transfected with iSuRe-Cre and Cre-expressing plasmids, the LoxP-N-PhiM-pA-LoxP cassette is recombined/deleted and MbTomato-2A-Int-Cre is expressed. Scale Bars 55μm. Error bars indicate StDev. Source data are provided as a Source Data file

The limitations of the Cre/LoxP technology outlined above, are mainly related to the weak and often variable expression of promoters in transgenes containing the Cre or CreERT2 coding sequences, which frequently do not induce the deletion of all different types of floxed alleles in the cells where they are expressed (Fig. 1a, b). To overcome this, we sought to develop a new DNA construct that would be easy to induce by Cre/CreERT2-mediated recombination, and that would subsequently enable strong and sustained co-expression of a fluorescent reporter and a constitutively active Cre (Fig. 1c). We reasoned that this simple construct (*iSuRe-Cre*), when inserted in the genome of ES cells or mice, would significantly increase the efficiency and reliability of conditional genetic modifications in reporter-expressing cells. The *iSuRe-Cre* construct contains the ubiquitous and strong CAG promoter followed by a very short (1.1 kb), and therefore easy to recombine^13,14^, LoxP-flanked (floxed) DNA sequence. This sequence has the *N-PhiM* reporter gene, which encodes a non-cytotoxic and non-fluorescent mutated PhiYFP protein (Evrogen) having a nuclear localization signal^13,15^, and a transcription stop signal (Fig. 1c). After recombination/deletion of the floxed cassette by Cre or CreERT2, the *iSuRe-Cre* construct enables the strong co-expression of a bright membrane-localized fluorescent reporter (MbTomato) and a constitutively active and permanently expressed Cre protein. These two proteins are separated by the viral 2 A peptide to guarantee equimolar expression^16^ and complete correlation between the MbTomato reporter expression and Cre activity (Fig. 1c).

Our first attempts to include these elements in the same plasmid failed. All plasmids containing the Cre-coding sequence had recombination/deletion of the floxed *N-PhiM-pA* cassette (Fig. 1d). This undesired recombination in *E. Coli* of plasmids containing Cre and floxed cassettes in *cis* is due to the low-level expression of the Cre sequence even in the absence of adjacent prokaryotic promoters. This may explain why similar DNA constructs and mouse lines have not been produced or published in the past. We noted that other laboratories encountered the same technical problem and overcame it by introducing an intron in the middle of the Cre sequence^17^. This modification disrupts the correct expression of Cre in splicing-incompetent prokaryotic cells but is compatible with expression in eukaryotic cells, which splice out the non-coding intron and therefore express Cre. When we inserted the intron-interrupted Cre sequence (Int-Cre), downstream of the short LoxP-flanked transcription stop cassette, we were able to recover *iSuRe-Cre* plasmids without any leaky recombination/deletion in *E. Coli* (Fig. 1e). To monitor CAG promoter expression in the absence of Cre activity, we immunostained cells for the N-PhiM protein. Expression of the MbTomato reporter allowed us to detect cells with induced Cre-dependent recombination and equimolar high Int-Cre expression (Fig. 1f).

### Transgenic and *ROSA26* targeted *iSuRe-Cre* ES cells and mice

**Fig. 2:**
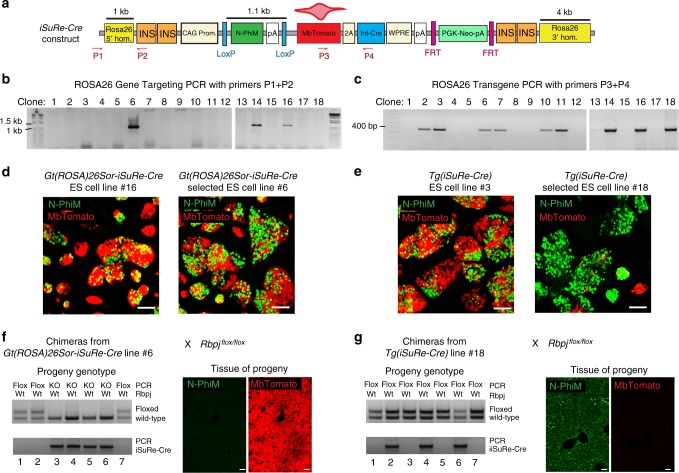
*iSuRe-Cre* DNA construct, ES cells, and mice. **a** *iSuRe-Cre* DNA construct used to produce gene-targeted or transgenic ES cells and mice. ROSA26 hom., ROSA26 locus homology arms; INS, chicken β-globin HS4 insulator sequence; FRT, short DNA sequences recognized by the recombinase Flp allowing deletion of the PGK-Neo selection cassette; P1-P4, primers used to genotype ES cells or mice. For further abbreviations and their definitions see also Fig. 1c legend. **b** PCR result with primers detecting the integration of the vector in the ROSA26 locus of ES cell clones 1–18. **c** PCR result with primers detecting the presence of the *iSure-Cre* allele in the genome of ES cells 1–18. **d** Representative images of different ROSA26-targeted (Gt(ROSA)26Sor) ES cell clones at baseline. N-PhiM is expressed if the allele is non-recombined, and MbTomato marks cells that had recombination of the construct without induction. Clone #6 has a higher proportion of non-recombined N-PhiM+ cells. **e** Representative images of different *Tg(iSuRe-Cre*) ES cell clones at baseline. Clone #18 has a higher proportion of non-recombined N-PhiM+ cells. **f** Mouse chimeras generated with Gt(ROSA)26Sor-iSuRe-Cre ES cell clone #6 were interbred with *Rbpj*^*flox/flox*^ animals, and the progeny were genotyped for the *Rbpj* and *iSuRe-Cre* alleles. All animals containing the *iSuRe-Cre* allele in the ROSA26 locus had deletion of the *Rbpj*-floxed allele and expressed the MbTomato reporter in all cells. **g** Mouse chimeras generated with *Tg(iSuRe-Cre)* ES cell clone #18 were interbred with *Rbpj* ^*flox/flox*^ animals, and the progeny were genotyped for the *Rbpj* and *iSuRe-Cre* alleles. Animals containing the *Tg(iSuRe-Cre)* allele did not have deletion of the *Rbpj-floxed* allele and did not express the MbTomato reporter. Instead they expressed the N-PhiM reporter due to the absence of Cre activity. Scale Bars 100 μm

The *iSuRe-Cre* DNA elements were finally inserted in a modified ROSA26 gene-targeting vector^12,18^ containing four chicken β-globin HS4 insulator sequences (Fig. 2a), to reduce genomic interference and silencing from neighbouring genomic regions^19^. The assembled plasmid was used to generate several independent embryonic stem (ES) cell lines (Fig. 2b, c). In some of the generated ES cell clones, the construct was inserted in the ROSA26 locus (*Gt(ROSA)26Sor-iSuRe-Cre*), while in others it was randomly inserted in the genome (*Tg(iSuRe-Cre)*). We observed a high degree of non-induced and sporadic recombination in most ES cell lines containing this construct in the genome (Fig. 2d, e), even in the absence of exogenous CreERT2 or Cre expression. The penetrance of this undesired recombination or leakiness was variable among clones and ranged from 15 to 95%. Although these results were disappointing, we proceeded to generate mice using the clone with the lowest degree of uninduced recombination from the *Tg(iSuRe-Cre)* and *Gt(ROSA)26Sor-iSuRe-Cre* ES cell lines (Fig. 2d, e, clones 6 and 18). The male chimeras generated with *Gt(ROSA)26Sor-iSuRe-Cre* ES cells carried the non-recombined allele in a large fraction of their cells, but the non-recombined allele was not passed on to any of their progeny. All progeny expressed the *MbTomato-2A-Int-Cre* cassette in all cells and had full deletion of the *Rbpj*-floxed allele (Fig. 2f), suggesting that when the iSuRe-Cre construct is targeted to the Rosa26 locus, the leakiness is high in the male germline, rendering the allele useless.

We next analysed the progeny of chimeras derived from *Tg(iSuRe-Cre)* ES cells with the construct integrated outside the ROSA26 locus. From these chimeras, we were able to obtain progeny carrying the *Tg(iSuRe-Cre)* allele and without *MbTomato-2A-Int-Cre* expression or recombination of th*e* *Rbpj*-floxed allele (Fig. 2g). Thus, the *Tg(iSuRe-Cre)* transgenic allele is not leaky in the male germline or in embryos, allowing us to generate a Cre/CreERT2-inducible dual Reporter-Cre-expressing mouse line.

### *iSuRe-Cre* is ubiquitously inducible and boosts recombination

**Fig. 3:**
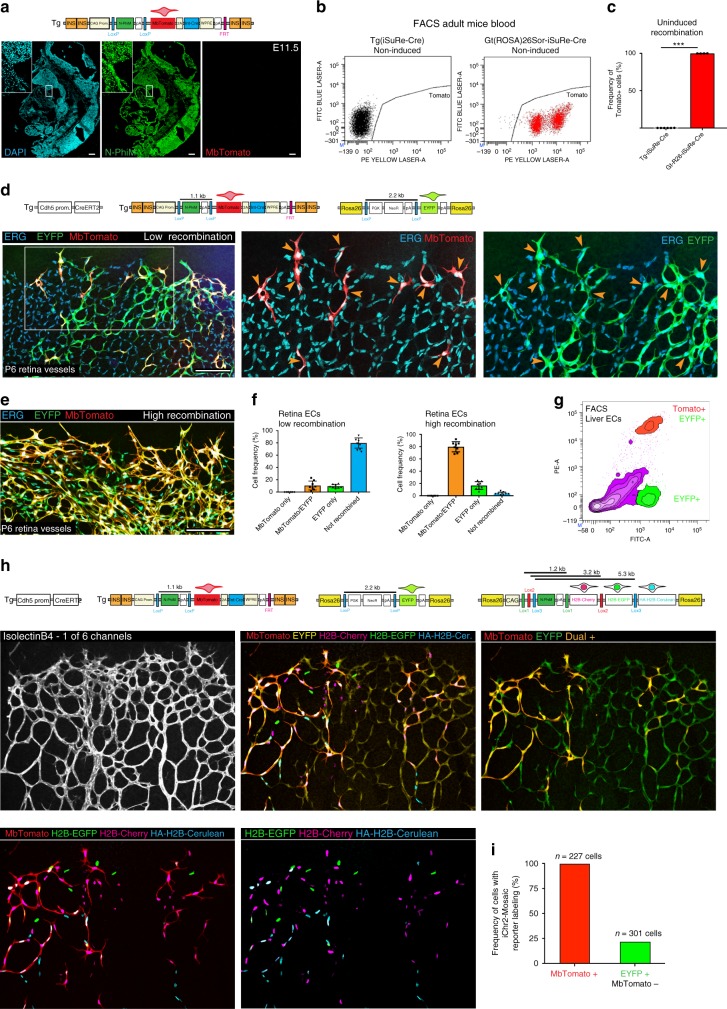
The *Tg(iSuRe-Cre)* allele is ubiquitously expressed and is a reliable reporter of recombination of other reporter alleles. **a** Analysis of *Tg(iSuRe-Cre)* expression in the absence of Cre activity reveals its expression (N-PhiM+) in most cells of mouse embryos. The allele does not self-recombine in embryos (MbTomato-negative). **b**, **c** FACS analysis reveals that the *Tg(iSuRe-Cre)* allele does not self-recombine in blood, unlike its ROSA26 gene-targeted version (each dot in the chart represents quantification of one animal). **d**, **e** Confocal micrographs of postnatal day (P) 6 retina vessels from animals with the alleles depicted above the panels and induced with tamoxifen from P1 to P3. All endothelial cells (ECs; nuclei, ERG+) expressing MbTomato-2A-Int-Cre also recombined the reporter allele *ROSA26*^*LSL-YFP*^. **f** Quantification of the different recombination events/reporters in retinal vessels with low and high frequencies of tamoxifen-induced CreERT2 recombination (*n* = 4 full retinas per group). **g** FACS analysis of liver ECs from tamoxifen-induced adult animals with the genotype indicated in d. All induced MbTomato+ cells also recombined the reporter allele *ROSA26*^*LSL-YFP*^. The MbTomato reporter from the *Tg(iSuRe-Cre)* allele is easier to separate from baseline autofluorescence. **h**, **i** Six-channel confocal micrograph of P6 retina vessels from animals (*n* = 3) with the genotype indicated above panels and induced with tamoxifen at P3. All cells expressing the MbTomato reporter recombined the two reporter alleles (*ROSA26*^*LSL-YFP*^ and *ROSA26*^*iChr2-Mosaic*^), resulting in expression of EYFP and one of the three possible nuclear-localized proteins (H2B-Cherry, H2B-EGFP, or HA-H2B-Cerulean). In contrast, only a small fraction of EYFP+ cells recombined and expressed this reporter in their chromatin/nuclei. Scale Bars 150 μm. Error bars indicate StDev; ***p < 0.0001; Two-tailed unpaired *t*-test (3c). Data in 3i indicate the mean frequencies obtained in the indicated cell groups

Transgenic alleles can be silenced in some cell types^20^, reducing their usefulness as research tools. The *iSuRe-Cre* transgene contains four chicken β-globin HS4 insulator sequences (Fig. 2a), to reduce genomic interference and silencing from neighbouring genomic regions^19^. However, insulated transgenic alleles can still be affected by the surrounding genome and chromatin status, which varies between cell types and across different developmental stages^21^. Therefore, we analysed expression of N-PhiM (reporter of transgene promoter activity) and MbTomato (reporter of transgene recombination and Int-Cre expression) in most embryonic and adult tissues of *Tg(iSuRe-Cre)* mice. This analysis revealed that the *Tg(iSuRe-Cre)* allele was not silenced and did not self-recombine in the embryo or in most organs and cell types (Fig. 3a–c and Supplementary Fig. 1a–e, g and i). The exception was adult and quiescent myocytes that form the skeletal and cardiac striated muscle tissue; a fraction of these cells consistently had leaky recombination of the *Tg(iSuRe-Cre)* allele and MbTomato expression (Supplementary Fig. 1f, h, i). Since the purpose of this line is the performance of tissue-specific and inducible gene loss-of-function experiments, which should yield a phenotype only after induction, the leaky expression and recombination in a fraction of quiescent adult myocytes will not affect the utility of the line for manipulating and understanding gene function during embryonic development or in other cell types in adults. Echocardiography analysis of 3-month-old adult mice revealed no significant differences in heart function (Supplementary Table 1). All animals containing the *Tg(iSuRe-Cre)* allele and singly or compound homozygous for different floxed alleles (*Dll4, Kdr, Rbpj, Fgfr1, Fgfr2, Myc, Mycn, Hif1*, or *Hif2*) were born at Mendelian rates, were healthy, and had normal behaviour (Supplementary Fig. 1j), indicating that the spontaneous deletion of these genes in a fraction of quiescent adult myocytes is not deleterious.

We next evaluated the relative inducibility rate of the *Tg(iSuRe-Cre)* allele by combining it with *Cdh5-CreERT2* and other available Cre-reporter alleles, such as the *Gt(ROSA)26Sor-LoxP-STOP-LoxP-EYFP* allele (abbreviated here as *ROSA26*^*LSL-EYFP*^)^11^. At both low or high recombination frequencies, all MbTomato+ cells were EYFP+ (Fig. 3d, e), demonstrating the high recombination efficiency in MbTomato-2A-Cre+ cells. The inducibility of the *Tg(iSuRe-Cre)* allele by CreERT2, was similar to the *ROSA26*^*LSL-EYFP*^ allele at low doses of tamoxifen, even if not targeted to the *ROSA26* locus (Fig. 3f), likely due to the shorter genetic distance between the LoxP sites flanking the N-PhiM-pA cassette (1.1 kb vs 2,2 kb)^13,14^. In addition, inclusion of the stronger CAG promoter, the brighter fluorescent protein (Tomato), and the WPRE element (Fig. 2a) results in higher reporter expression in each recombined cell, making it easier to detect and distinguish from background tissue autofluorescence than the other commonly used ROSA26 reporter allele (*ROSA26*^*LSL-EYFP*^), as determined by FACS (Fig. 3g)^11^. To compare the reliability of the *Tg(iSuRe-Cre)* and *ROSA26*^*LSL-EYFP*^ alleles in reporting cells with other alleles recombination, we intercrossed animals having the *Tg(iSuRe-Cre)* and *ROSA26*^*LSL-EYFP*^ alleles with animals having the *ROSA26*^*iChr2-Mosaic* 13^ reporter allele (Fig. 3h). This allele contains three distinct and mutually exclusive LoxP sites that compete for the recombination event, lowering its recombination efficiency (Supplementary Fig. 2a). Once the *ROSA26*^*iChr2-Mosaic*^ allele is recombined, cells express chromatin/nucleus-localized proteins (H2B-Cherry, H2B-GFP, or HA-H2B-Cerulean) that can be clearly distinguished from MbTomato or EYFP (Fig. 3h). Whereas only 23% of single EYFP+ cells had also recombined the *ROSA26*^*iChr2-Mosaic*^ allele, all individual cells expressing the *MbTomato-2A-Int-Cre* cassette had recombined the *ROSA26*^*LSL-EYFP*^ and *ROSA26*^*iChr2-Mosaic*^ reporter alleles (Fig. 3h, i), demonstrating the high recombination efficiency in cells with activation of the *Tg(iSuRe-Cre)* allele. Collectively, these results show that the *Tg(iSuRe-Cre)* allele is expressed and inducible in most tissues and that it is also a much more reliable reporter of cells with recombination of other floxed alleles.

### *iSuRe-Cre* reliably reports cells with full gene deletion

**Fig. 4:**
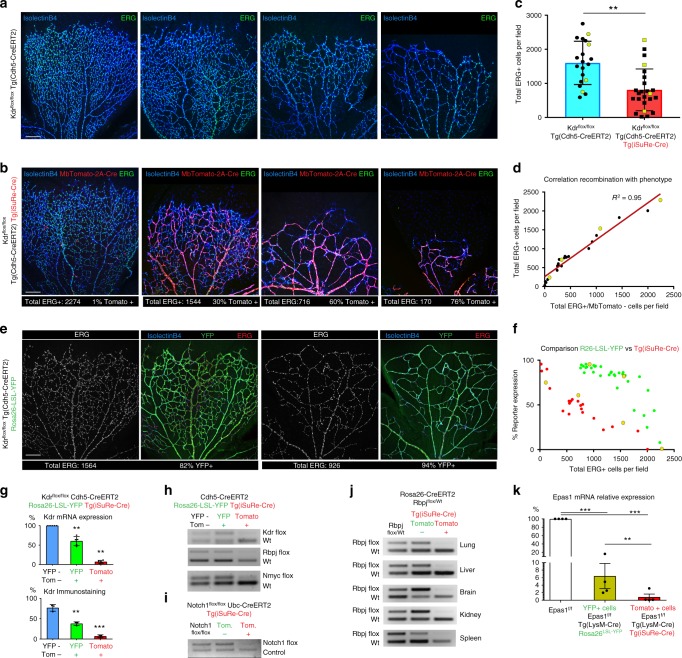
The *Tg(iSuRe-Cre)* allele increases the efficiency of inducible genetic modifications. **a**–**c** Representative confocal micrographs of P6 retina vessels labelled with IsolectinB4 (endothelial surface) and anti-ERG antibody (endothelial nuclei), obtained from animals with the genotype indicated to the left and induced with high-dose tamoxifen from P1 to P3. Images represent the observed phenotypic variability; EC number for each image is depicted in chart **c** (yellow dots). 20 vs 24 retina microscopic fields were analysed (*n* = 5 vs *n* = 6 animals per group). **d** Linear regression showing the high correlation (r^2^) between total Erg + EC number (phenotype) and the number of Erg + /MbTomato- cells. **e**, **f** Representative confocal micrographs of P6 retina vessels from animals with the genotype indicated to the left and induced with high-dose tamoxifen from P1 to P3. Images represent the phenotypic variability of animals with the same genotype. EC number and reporter expression frequency is indicated below the figures. A comparison of EC number and reporter expression frequency is depicted in chart for 24 vs 34 retina microscopic fields (*n* = 6 animals per group). Yellow dots in chart f represent the values for images in b and e.  **g** qRT-PCR analysis of Kdr mRNA levels from FACS-sorted liver ECs (*n* = 5 animals), or immunostaining analysis of liver sections of animals (*n* = 3) with the indicated genotype (see also Supplementary Fig. 2b). **h** Semi-quantitative competitive PCR showing the efficiency of *Kdr, Rbpj* and *Nmyc* deletion in FACS-sorted cells of adult mice with the indicated genotypes and induced with tamoxifen. Note that some cross-contamination of samples and DNA may occur during tissue dissociation and FACS of mutant and wild-type cells. **i** Semi-quantitative competitive PCR for the *Notch1* floxed allele and a control genomic sequence showing the efficiency of the *Ubc-CreERT2* induced *Notch1* deletion in Tomato+ and Tomato- cells of the liver. **j** Semi-quantitative competitive PCR showing the efficiency of *Rbpj* gene inducible deletion in the Tomato− and Tomato+ cells of several distinct organs from *Rosa26-CreERT2* mice induced with tamoxifen. **k** *Epas1* mRNA relative levels (qRT-PCR) in *LysM-*Cre-reporter-expressing bone marrow-derived macrophages (*n* = 4). Scale Bars 200 μm. Error bars indicate StDev; **p < 0.001; ***p < 0.0001. Two-tailed unpaired *t*-test (4c) or ANOVA (4 g and 4k). Source data are provided as a Source Data file

Having confirmed the *Tg(iSuRe-Cre)* allele as a bona fide recombination reporter, we next sought to determine if it would also allow us to achieve higher efficiencies of CreERT2-inducible gene deletion. In contrast to the very short (1.1 kb), inter-loxP genetic distance in the *Tg(iSuRe-Cre)* allele, the floxed alleles of many other genes have significantly larger genetic distances and require two allelic recombination events for full gene loss-of-function. To determine the robustness of the *Tg(iSuRe-Cre)* allele in inducing genetic deletions, we generated mice containing the *Cdh5-CreERT2* and *Tg(iSuRe-Cre)* transgenes and two *Kdr* floxed (*Kdr* ^*flox*^) alleles^22^, where the LoxP sites are 4.7 kb apart. *Kdr* is an essential gene for EC differentiation, proliferation, migration, and survival^23,24^. The results show that when *Kdr*^*flox/flox*^ *Cdh5-CreERT2*^*Tg/Wt*^ mice contain the *Tg(iSuRe-Cre)* allele, there is a significant decrease in the number of endothelial cells (ERG + nuclei), particularly in retinas with higher induction of the *Tg(iSuRe-Cre)* allele (Fig. 4a–c). These results also show that full *Kdr* recombination and loss-of-function cannot be consistently achieved with tamoxifen-inducible conditional genetics unless the *Tg(iSuRe-Cre)* allele is present. As with any tamoxifen induction experiment, there was some littermate variability in the degree of recombination of the *Kdr*^*flox*^ and *Tg(iSuRe-Cre)* alleles. However, the mutants with almost complete expression of the *MbTomato-2A-Int-Cre* cassette could be safely selected for phenotypic and statistical analysis (Fig. 4d). In animals containing the *ROSA26*^*LSL-YFP*^ reporter allele, the number of retina ECs at P6 was significantly higher, even in tissues with very high reporter recombination rates (Fig. 4e, f). Importantly, the vascular phenotype obtained at high *ROSA26*^*LSL-YFP*^ reporter recombination rates was not representative of the real full gene loss-of-function phenotype, reflecting the poor correlation between the recombination of a conventional Cre-reporter (i.e. *ROSA26*^*LSL-YFP*^) and deletion of the gene of interest. In addition, the detected endpoint frequency of YFP+ cells was on average significantly higher than the frequency of Tomato+ (iSuRe-Cre+) cells in P6 retina vessels (Fig. 4f). This is in agreement with the fact that *Kdr* is an essential gene for EC proliferation and survival^23,24^, therefore the initially (P1–P3) recombined mutant cells are strongly outcompeted by the remaining *Kdr* wild-type cells over time, resulting on average in a relatively lower frequency of Tomato+ cells, because these efficiently delete Kdr, unlike the YFP+ cells (Fig. 4g and Supplementary Fig. 2b). Importantly, retinas with around 50–60% *iSuRe-Cre*/Tomato+ cells at P6 (Fig. 4b), showed more severe vascular defects than retinas with 80–95% *ROSA26*^*LSL-YFP*^/YFP+ cells (Fig. 4e).

Besides the gene *Kdr*, we also examined the efficiency of the *Tg(iSuRe-Cre)* allele in deleting the genes *Rbpj*, *Mycn*, and *Notch1*. Cells expressing MbTomato had very efficient deletion of these genes (Fig. 4h and Supplementary Fig. 2c, 2d). Importantly, a significant fraction of cells expressing a conventional Rosa26 Cre-reporter (YFP+), had no deletion of these genes (Fig. 4h).

To analyse the efficiency of the *Tg(iSuRe-Cre)* allele in the recombination of genes, in other cell types and organs, we interbred this mouse line with the ubiquitously expressed *Rosa26-CreERT2*^25^ and *Ubc-CreERT2* lines^26^, and induced recombination of the genes *Rbpj* and *Notch1* with tamoxifen. In cells with *Tg(iSuRe-Cre)* allele induction (MbTomato+), the *Rbpj* and *Notch1* genetic deletion was highly efficient (Fig. 4i, j).

In addition to inducible CreERT2 mouse lines, we also compared gene-deletion efficiency of the *Tg(iSuRe-Cre)* allele versus a conventional *ROSA26*^*LSL-YFP*^ allele when these lines are interbred with the constitutively active LysM-Cre line, that is specifically expressed in granulocytes and macrophages^27^. In contrast to inducible CreERT2, Cre usually recombines most floxed alleles in cells in which it is expressed; however, this may vary with the levels of Cre expression per cell. We found that while deletion of the floxed gene *Epas1* is incomplete in LysM-Cre + YFP + cells, it is complete in LysM-Cre + MbTomato-2A-Int-Cre+ cells (Fig. 4k).

### *iSuRe-Cre* enables deletion of multiple genes and epistasis

A common strategy for understanding and validating genetic redundancy or gene interaction networks is to induce the deletion of two or more genes in the same tissue and analyse if deletion of one gene aggravates or rescues the phenotype caused by the other. This is a fundamental method for understanding how genes interact to regulate a given biological process.

**Fig. 5:**
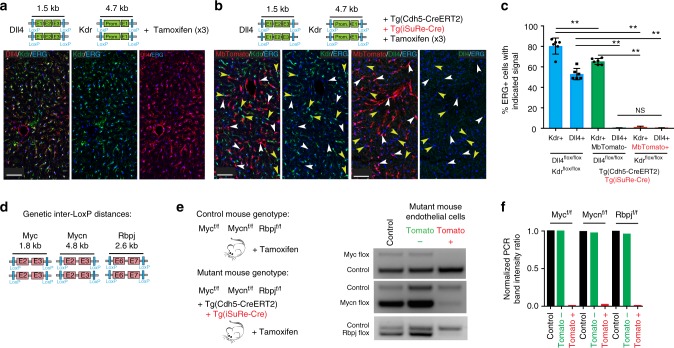
The *Tg(iSuRe-Cre)* allele enables multiple gene deletions in single cells or tissues. **a** The schemes illustrate the *Dll4* and *Kdr* floxed alleles, showing inter-LoxP-site genetic distance, which is significantly larger in the *Kdr* allele. All four alleles must be deleted to achieve full dual gene loss-of-function. Kdr and Dll4 proteins are expressed in most liver ECs (ERG+, nuclei) of *Dll4*^*flox/flox*^*/Kdr*^*flox/flox*^ animals injected with tamoxifen on 3 consecutive days. **b** Adult mice carrying in addition the *Tg*(*Cdh5-CreERT2)* and *Tg(iSuRe-Cre)* alleles and treated with the same high-dose tamoxifen for 3 consecutive days show very pronounced deletion of *Dll4*, but not *Kdr*, in liver MbTomato^-^/ERG + ECs (yellow arrowheads). However, MbTomato^+^ cells (white arrowheads) have complete deletion of both genes. **c** Quantification of the immunostaining signals for ERG, Dll4, Kdr, and MbTomato in large liver sections of the indicated animals. **d** Illustration of the *Myc*, *Mycn*, and Rbpj-floxed alleles showing the genetic distances between the LoxP sites. **e** Genotypes of control and mutant adult mice injected once with 1 mg of tamoxifen and used for gene-deletion quantification by PCR. The control PCR band provides a DNA input quantitative control for the PCR, since it corresponds to a wild-type genomic sequence, present in all DNA samples (see supplementary table 2 for primer sequences). Animals containing the *Tg*(*Cdh5-CreERT2)* and *Tg(iSuRe-Cre)* alleles have deletion of the six floxed alleles only in FACS-sorted MbTomato^+^ cells, as detected by semi-quantitative competitive PCR. Weak *Mycn* and *Rbpj*-floxed bands in the MbTomato + sample PCR may result from incomplete gene-deletion or contamination of this sample with MbTomato-negative cells, or their DNA, during the FACS protocol. **f** Image J quantification of the relative intensity of the floxed and control PCR gel bands shown in **e**, providing an estimate of the degree of the indicated floxed gene deletion. Scale Bars 65 μm. Error bars indicate StDev. **p < 0.001. NS nonsignificant. One-way ANOVA with Tukey’s post hoc test. Source data are provided as a Source Data file

Given the stronger Cre activity and recombination efficiency shown by the *Tg(iSuRe-Cre)* allele, we reasoned that it would be particularly useful in inducing and reporting cells with full deletion of multiple genes or floxed alleles. As an example, we present data for the simultaneous deletion of the genes *Dll4/Kdr* and *Myc/Mycn/Rbpj* in ECs expressing *Cdh5-CreERT2*, after inducing animals with tamoxifen. This implies the deletion of four or six floxed alleles in each cell to achieve cell-autonomous multiple gene loss-of-function and in this way perform epistasis analysis. Dll4 and Kdr are expressed by most liver ECs, and their presence in ECs could be detected by immunostaining (Fig. 5a). In animals containing the *Cdh5-CreERT2* and *Tg(iSuRe-Cre)* alleles and injected three times with tamoxifen, we observed deletion of *Dll4* in most liver ECs, but not of *Kdr* (Fig. 5b, c). This result shows how recombination efficiency differs among different floxed genes. Importantly, MbTomato + ECs had complete deletion of both *Dll4* and *Kdr*, whereas most MbTomato-negative cells maintained *Kdr* expression, even after three high-dose tamoxifen injections (Fig. 5b, c).

We next analysed multiple gene-deletion efficiency in *Myc/Mycn/Rbpj*^*flox/flox*^, *Tg(Cdh5-CreERT2),* and *Tg(iSuRe-Cre)* mice induced with a single 1 mg injection of tamoxifen (Fig. 5d, e). Technically, it is much more difficult to confirm the simultaneous deletion of more than two genes in single cells by direct immunostaining, due to the lack of compatible commercial antibodies to distinguish all epitopes of the encoded proteins in combination with other tissue or reporter markers. We therefore extracted DNA from FACS-sorted MbTomato+ and MbTomato− ECs (CD31+) isolated from the induced animals and assessed gene deletion by semi-quantitative PCR. Only MbTomato-2A-Int-Cre+ cells had deletion of all three genes (Fig. 5d–f). The *Tg(iSuRe-Cre)* allele enabled us to achieve high efficiency in multiple gene deletion and safely correlate the expression of the single and easy-to-detect MbTomato reporter with the deletion of three genes (six floxed alleles), which encode proteins that could not be detected by tissue immunostaining. A potential disadvantage of the *Tg(iSuRe-Cre)* allele to induce multiple floxed alleles deletion could be the occurrence of interchromosomal recombination events between different floxed genes, located in different chromosomes. However, interchromosomal recombination is far less frequent than intrachromosomal recombination in vivo^28^. We could not detect by PCR interchromosomal recombination events (Supplementary Fig. 3a).

### *iSuRe-Cre* is not leaky when interbred with other Cre lines

Tissue-specific Cre mouse lines, normally express Cre at high levels in a given tissue in which the transgene promoter is active, but they may also express it at low levels in other undesired tissues, and this can be enough to recombine the most sensitive floxed genes or Cre activity reporters localized in the ROSA26 locus.

**Fig. 6:**
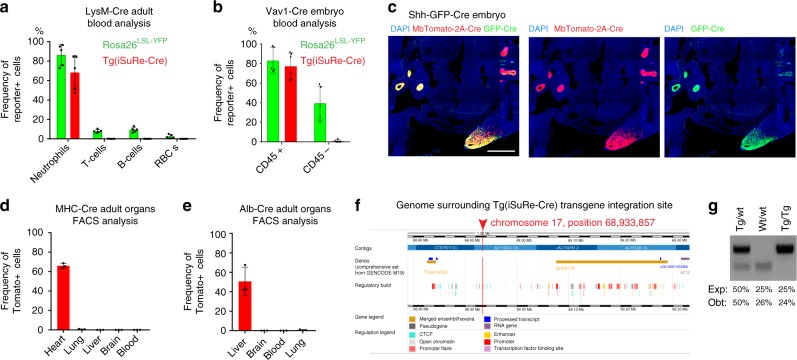
The *Tg(iSuRe-Cre)* allele is not leaky. **a**, **b** Charts showing the comparison of the frequency of cells with YFP or MbTomato-2A-Int-Cre expression when the *Tg(iSuRe-Cre)* or *ROSA26*^*LSL-YFP*^ reporter lines are intercrossed with the indicated Cre lines (*n* = 5 animals or embryos per group). LysM-Cre and Vav1-Cre lines both recombine the *ROSA26*^*LSL-YFP*^ reporter in non-desired cell types, but not the *Tg(iSuRe-Cre)* allele. **c** Confocal micrographs showing the recombination of the *Tg(iSuRe-Cre)* allele in Shh-GFP-Cre embryos. **d**, **e** The *Tg(iSuRe-Cre)* allele is not leaky and is induced only in the desired cell types/organs when the line is interbred with tissue-specific Cre-expressing lines (*n* = 3 animals per line). **f** Example readout illustrating the genes and regulatory regions surrounding the transgene integration site on chromosome 17. **g** Genomic DNA PCR detection of homozygous animals, found at the expected Mendelian ratio (*n* = 79). Error bars represent StDev. Scale bar 350 μm. Error bars indicate StDev. Source data are provided as a Source Data file

Given the high sensitivity of the *Tg(iSuRe-Cre)* allele to *Cdh5-CreERT2* induced recombination (Fig. 3f), and its location outside the ROSA26 locus, we next analysed if the combination of this allele with other constitutive Cre-expressing alleles could result in non-specific recombination or leakiness. This analysis revealed that the *Tg(iSuRe-Cre)* allele is not leaky in the different cell types, organs and developmental contexts analysed. With *LysM-Cre*^27^ and *Vav1-Cre*^29^, recombination of the *Tg(iSuRe-Cre)* allele was detected only in the expected fraction of haematopoietic lineages (Fig. 6a, b). Interestingly, we detected non-specific recombination of the *ROSA26*^*LSL-YFP*^ allele with these two Cre lines (Fig. 6a, b), in line with earlier reports^7,30^. Embryos containing the *Shh-GFP-Cre* allele had expression of the transgene in GFP-Cre+ endoderm derived cells and limb buds (Fig. 6c). Finally, when combined with the *Tie2-Cre*, *Alb-Cre*, and *MYHC-Cre* alleles, the *Tg(iSuRe-Cre)* transgene was also specifically expressed in blood or endothelial cells (Tie2+), liver hepatocytes (Alb+), and heart cardiomyocytes (MyHC+), respectively (Fig. 6d, e and Supplementary Fig. 3b, 3c). Together, these results indicate that the genomic integration site of the *Tg(iSuRe-Cre)* allele is leaky in only a small fraction of adult myocytes (Supplementary Fig. 1), even when combined with other tissue-specific Cre-expressing transgenes.

Importantly, we were able to map the transgene integration site to a non-coding region between genes *Tmem200c* and *Epb41I3* (Fig. 6f) in chromosome 17 (Cergentis). This allowed us to design primers to detect homozygous animals, and confirm that they are born at the expected Mendelian ratio (Fig. 6g), indicating that transgene integration did not disrupt any important regulatory elements. This genomic region will be of interest for gene targeting of other similar constructs, since it escapes the Rosa26-associated leakiness in the mouse germline (Figs. 2f and 3b, c), while, like the Rosa26 locus, permitting the expression of the integrated transgenes in all the analysed developmental stages and organs.

### Permanent expression of Cre from the *iSuRe-Cre* is non-toxic

High levels of Cre expression, or induction of CreERT2 with high doses of tamoxifen, has been shown to cause cellular toxicity and induce unexpected cellular phenotypes^4^. Injection, transfection, or infection of cells with Cre-expressing DNA constructs, leading to multicopy epissomal or multicopy integration in the same genome locus, frequently generates high Cre expression levels and cellular toxicity^4,31–33^. In vivo, Cre toxicity was reported for the *Rosa26-CreERT2*^4,33^ and *α-MHC-MerCreMer*^34,35^ mouse lines, when high tamoxifen doses were used, and also for the *α-MyHC-Cre*^36^ mouse line, which shows minor cardiac defects in adult mice from 3 months of age that, become more pronounced at 6 months^36^. In contrast to these alarming reports, cell toxicity has not been reported for the large majority of the more than 300 Cre-expressing mouse lines that have been generated and characterized (Jackson laboratory). All these lines have tissue-specific promoters driving continuous expression of Cre, in the target tissue. These include numerous lines with multicopy integration of Cre-expressing transgenes, such as the 10 copies in Alb-Cre mice^37^ and the 20 copies in Tie2-Cre mice^38^.

**Fig. 7:**
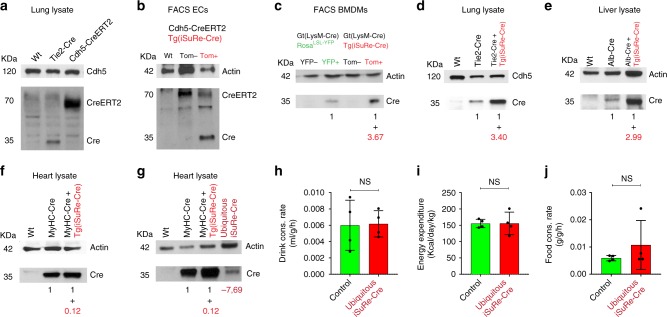
Comparison of Cre expression levels in different tissues and mouse lines. **a** Western blot analysis of lung lysates from mice with the indicated genotypes, showing Cre (35kDa) and CreERT2 (70 kDa) expression differences in Tie2-Cre and Cdh5-CreERT2 mouse lines. **b** Western blot analysis of FAC-sorted endothelial cells (CD31-APC+) from the indicated mice, showing Cre (35kDa) and CreERT2 (70 kDa) expression in wild-type, MbTomato+ and MbTomato− cells. **c** Western blot analysis of FAC-sorted bone marrow-derived macrophages (BMDMs) obtained from the indicated mice. **d**, **e** Western blot analysis of lung and liver lysates from mice with the indicated genotypes, showing the increase in Cre protein levels in tissues of animals with expression of the *Tg(iSuRe-Cre)* allele. **f** Western blot analysis of heart lysates from mice with the indicated genotypes, showing no significant increase in Cre protein levels in tissues of *MyHC-Cre* *+* *Tg(iSuRe-Cre)* animals. **g** Western blot analysis of heart lysates showing that animals with recombination of the *Gt(Rosa)26Sor-iSuRe-Cre* allele in the germline, and with ubiquitous iSuRe-Cre expression in their hearts (not only MyHC-Cre + cardiomyocytes), have significantly lower Cre levels than MyHC-Cre + hearts. **h**–**j** Metabolic studies in adult littermate mice (9 months old, *n* = 4 per group) having or lacking ubiquitous expression of the *Gt(Rosa)26Sor-iSuRe-Cre* allele. Values in red below the blots indicate the fold change in Cre protein relative to Tg(iSuRe-Cre) allele expression. Error bars indicate StDev; NS nonsignificant. Two-tailed unpaired *t*-test. Source data are provided as a Source Data file

The *Tg-iSuRe-Cre* allele integrated in chromosome 17, and is expressed as a single copy, which may result in weaker expression compared with other tissue-specific Cre mouse lines containing several copies of the Cre transgene. Although the expression of the *Tg-iSuRe-Cre* allele is driven by the strong CAG promoter, and enhanced by the WPRE element (Fig. 2a), the included 2A peptide decreases overall translation efficiency^13^. Therefore, instead of comparing RNA expression levels, we decided to compare Cre protein expression levels in cells expressing the *Tg(iSuRe-Cre)* allele and other existing Cre alleles, in order to assess potential cell toxicity related to high Cre expression. The levels of Cre expression driven by the Cre-induced *Tg(iSuRe-Cre)* allele, were within the normal range, providing a moderate increase in Cre protein levels in the tissues expressing Cdh5-CreERT2 (endothelial cells), LysM-Cre (macrophages), Tie2-Cre (endothelial and blood cells), and Alb-Cre (hepatocytes) transgenes (Fig. 7a–e). Importantly, comparison of Cre expression in the hearts of *MyHC-Cre* and *MyHC-Cre Tg(iSuRe-Cre)* mice, did not reveal any significant increase in Cre protein levels (Fig. 7f). We also analysed Cre levels in the hearts of animals with germline recombination of the *Gt(ROSA)26Sor-iSuRe-Cre* allele (ubiquitous), that do not contain the *MYHC-Cre* allele, but have expression of Cre in all cardiomyocytes and all other heart cells (i.e. vascular cells, fibroblasts, and blood cells). In these animals, cardiac Cre expression driven by the CAG promoter was much lower than that of the *MyHC-Cre* transgene (Fig. 7g). The very high expression levels of the 6-copy *MyHC-Cre* transgene may explain the cardiotoxicity detected in 3–9-month-old *MyHC-Cre* mice^36^.

Further supporting the non-toxicity of iSuRe-Cre expression, mice with germline recombination of the *Gt(ROSA)26Sor-iSuRe-Cre* allele (Fig. 2f) are alive and fertile. Expression of MbTomato-2A-Int-Cre in all cells from conception and, throughout embryonic, postnatal, and adult life was not deleterious. In addition, metabolic studies of 7-month-old mice did not reveal any significant differences (Fig. 7h–j). We therefore conclude that expression of MbTomato-2A-Int-Cre driven by the single copy CAG promoter is non-toxic.

## Discussion

Our study reinforces the notion that the methods commonly used to induce and report gene loss-of-function in mice are often inefficient and unreliable^4,7^. This is especially problematic for genes that are difficult to delete or encode proteins whose absence cannot be clearly detected in multicellular tissues. In conditional genetics, fluorescent Cre-reporter-expressing cells are usually considered mutant cells, even though a variable fraction of them may escape the desired gene deletion, especially if this gives them a competitive advantage (Fig. 4e, f)^39^. Many Cre transgenes are sensitive to epigenetic regulation, which causes variegation or weak expression in a fraction of cells^7^. We show several examples in which weak Cre or CreERT2 expression/activity may be enough to recombine a floxed reporter allele localized in the permissive Rosa26 locus; however, this weak activity is insufficient to fully delete other genes, that require higher or longer-lasting Cre expression, which can be provided by the *iSuRe-Cre* allele (Supplementary Fig. 4a). This work also shows that a conventional Cre-reporter allele, only accurately reports recombination of itself, not of other reporters or other floxed genes. In contrast to conventional Cre reporters, the *iSuRe-Cre* allele accurately reports cells with recombination/deletion of any other tested reporter or floxed gene, greatly decreasing the rate of false positives.

Like any other conventional Cre-reporter, the *iSuRe-Cre* allele does not prevent the occurrence of false-negatives, since some reporter-negative cells may show deletion of other floxed genes that are easier to recombine. However, we show that with the exception of the gene *Dll4*, all other tested floxed genes (*Kdr, Myc, Mycn, Notch1, Rbpj*) were only efficiently deleted in reporter-expressing cells (Supplementary Fig. 4b). Therefore, gene-deletion false-negatives do not occur at a significant level with the *iSuRe-Cre* or other conventional Cre-reporters, except for the few genes in which deletion of two gene alleles, is easier to induce than the recombination of a single copy of these reporter alleles.

High levels of Cre expression has been shown to cause cellular toxicity and induce unexpected cellular phenotypes^4^, which may be a disadvantage of the *iSuRe-Cre* allele (Supplementary Fig. 4c). We have not observed toxicity in cells or animals expressing the *iSuRe-Cre* allele permanently since conception. This is likely because its expression is not much higher than other existing Cre lines (Fig. 7). However, in order to have a complete control of conditional genetic experiments, animals having induction of the *iSuRe-Cre* allele and lacking other floxed genes can be analysed.

Multiple gene deletion in the same cell or tissue is frequently required to determine epistasis or functional genetic interactions. The use of the new *iSuRe-Cre* allele will allow the analysis of phenotypes produced by multiple gene deletions, with high cellular resolution and genetic reliability in live or fixed tissues. Efficient genetic deletions can also be achieved in a mosaic fashion in single cells, by titrating the CreERT2 ligand tamoxifen, because gene deletion per MbTomato-2A-Int-Cre+ cell is highly efficient, even if the initial CreERT2-dependent recombination induction frequency is very low.

In summary, we propose use of the *iSuRe-Cre* mouse line instead of conventional Cre activity reporters, which we (Figs. 3 and 4) and others^4,6^ have demonstrated are not bona fide reporters of the recombination/deletion of other genes. Moreover, unlike other binary FlpO/FlpOERT2-inducible Cre-expressing systems^40^, the *iSuRe-Cre* allele is not leaky in the male germline and is fully compatible with the numerous existent LoxP and Cre/CreERT2 alleles^1–3^. This new genetic tool will significantly increase the ease, efficiency, and reliability of conditional genetic modifications in the mouse, the most widely used model organism in biomedical research.

## Methods

### Mice

For the generation of transgenic mice, several mouse ES cell clones were generated and genotyped (Fig. 2) to identify clones with ROSA26 gene-targeting or transgenesis of the *iSuRe-Cre* construct (see ES cell culture method below). With the selected ES cell clones, several mouse chimeras with high germline transmission rates were obtained and used to produce the mouse lines *Gt(ROSA)26Sor-iSuRe-Cre* and *Tg(iSuRe-Cre)*. In addition, we interbred *Tg(iSuRe-Cre)* mice with the following mouse lines: *Tg(Tie2-Cre)*^38^*, Gt(Shh-GFP-Cre)*^41^*, Tg(Vav1-Cre)*^29^*, Tg(Alb-Cre)*^37^*, Tg(MyHC-Cre)*^42^*, Gt(LysM-Cre)*^27^*, Tg(Ubc-CreERT2)*^26^*, Gt(ROSA)26-CreERT2*^25^*, Tg(Cdh5-CreERT2)*^43^, *Gt(ROSA)26*^*LSL-EYFP*^ ^11^, *Gt(ROSA)26*^*iChr2-Control-Mosaic*^ ^13^, *Kdr*^*flox/flox*^ ^22^, *Dll4*^*lox/flox*^ ^44^, *Notch1*^*flox/flox*^ ^45^, *Rbpj*^*flox/flox*^ ^46^, *Epas1*^*flox/flox*^ ^47^, *Myc* ^*floxed*^ ^48^, and *Mycn*^*floxed*^ ^49^. To activate recombination in animals containing CreERT2 alleles, tamoxifen (Sigma) was injected in pups or adult mice at the indicated stages at a dose of 40 μg/g body weight. Genotyping primers are listed in Supplementary table 2. Experiments involving animals were conducted in accordance with official guidelines and laws, following protocols approved by local animal ethics committees and authorities (Comunidad Autónoma de Madrid and Universidad Autónoma de Madrid—CAM-PROEX 177/14 and CAM-PROEX 167/17). Male and female mice were used for the analysis and maintained under specific pathogen-free conditions.

### ES cell culture and genotyping

The basic elements of the *iSuRe-Cre* DNA constructs (Fig. 1 and Fig. 2) were obtained from varied sources and assembled by standard DNA cloning^13^. The Int-Cre sequence was obtained from plasmid p210 pCMV-CREM^17^, a gift from Jeffrey Green (Addgene plasmid #8395).

Mouse ES cells on the G4 background^50^ were cultured in standard ES cell culture medium (DMEM containing Glutamax (31966–047, Gibco), 15% FBS (tested for germline transmission), 1x NEAA (Hyclone, SH3023801), 0,1% ß-mercaptoethanol (Sigma, M7522), 1x Pen/Strep (Lonza, DE17-602E) and LIF) in dishes covered with a feeder layer of mouse embryonic fibroblasts (MEFs). For classical gene targeting with the large *iSuRe-Cre* plasmid, 25 ug of linearized DNA was used to electroporate 5 million ES cells. Selection in 200 ug/ml G418 (Geneticin) was performed for 6 days, after which individual colonies were picked for storage, PCR, and Southern blot screening. Selected positive clones were expanded and used for microinjection in host blastocysts of the C57Bl/6 J strain. Chimeras with a high percentage of agouti coat color were then crossed with mice to obtain germline transmission of the targeted insertion. PCR with ROSA26 5’homology arm flanking primers (Supplementary table 2), allowed us to identify ES cell clones with precise homologous recombination and insertion of the *iSuRe-Cre* construct in the *ROSA* locus. After identification of clones with precise gene targeting, we performed PCR with the MbTomato F and Cre R primers (Supplementary table 2) to detect ES cell clones containing the MbTomato-2A-Int-Cre transgene sequence. Selected ES cell clones were expanded for further analysis and mouse generation.

### Immunostaining

For ES cell immunostaining (Figs. 1 and 2), cells were fixed for 10 min in PBS containing PFA4% and Sucrose 4%. After a brief rinse in PBS, cells were permeabilized in 0.1% Triton for 10 min and then immersed in a blocking solution (10% Fetal bovine serum in PBS). Primary antibody (1:400, Rabbit Anti-PhiYFP, AB602, Evrogen) was diluted in blocking solution and incubated for 2 h at room temperature or overnight, followed by three washes in PBS of 10 min each and incubation for 1–2 h with conjugated secondary antibodies (1:400, Invitrogen or Biotium) at room temperature. After three washes in PBS, cells were mounted with Fluoromount-G (SouthernBiotech).

For immunostaining of mouse retinas (Figs. 3 and 4 and Supplementary Fig. 2, 3), eyes from mouse pups were dissected and fixed for 1 h in a solution of PFA4% in PBS. After washing the tissue in PBS twice, retinas were microdissected and processed for immunostaining following a very similar protocol previously described above for the cells. The only difference is that the blocking/permeabilization buffer contains 0.3% Triton (Sigma), 3% FBS, 3% Donkey Serum (Millipore), and antibody washes were more extended in time; on average for 30 min each. Biotinylated IsolectinB4 (1:50, Vector Labs B-1205) and Streptavidin-405 (1:400, Invitrogen, S-32351) were used to label the surface of the vessels. Conjugated rabbit anti-ERG-Alexa647 (1:200, Abcam, ab196149) was used to detect the endothelial nuclei, and mouse anti-HA-647 (1:300, Cell signalling, #3444) was used to detect HA-H2B-Cerulean + nuclei. The other endogenous fluorescent signals (H2B-EGFP, EYFP, MbTomato, and H2B-Cherry) were excited and scanned with different compatible laser lines and defined detectors (488 nm, 514 nm, 546 nm, and 595 nm, respectively).

For immunostaining of mouse embryos and organ sections (Figs. 2f, g, 3a, 5a, b, 6c and Supplementary Fig. 1 and Supplementary Fig. 2b) tissues were fixed for 2 h in a solution of PFA 4% in PBS at 4 °C. After washing the tissue in PBS three times, organs were stored overnight in 30% sucrose (Sigma) in PBS. Then, organs and embryos were embed in OCT^TM^ (Sakura) and frozen at −80 °C. Cryosections of organs (35 μm) and embryos (15 μm), were cut on a cryostat (Leica). Sections were washed three times 10 min each in PBS and blocked/permeabilized in PBS with 10% Donkey Serum (Milipore) and 1% Triton (organs) or 0,5% Triton (embryos). Primary antibodies were diluted in blocking/permeabilization buffer and incubated overnight at 4 °C. This step was followed by three washes in PBS of 10 min each and incubation for 2 h with conjugated secondary antibodies (1:400, Jackson Laboratories) and DAPI at room temperature. After three washes in PBS, cells were mounted with Fluoromount-G (SouthernBiotech). The following primary antibodies were used: rabbit anti-PhiYFP (1:200, Axxora, EVN-AB605); goat anti-Dll4 (1:200, R&D systems, AF1389); rat anti-VEGFR2 (1:200, BD Pharmingen, 550549), rabbit anti-Dsred (1:200, Clontech, 632496), and rabbit anti-ERG-Alexa647 (1:200, Abcam, Ab110639). To detect MbTomato in the same section as ERG, endogenous signals were scanned or rabbit anti-Dsred plus a Fab fragment CY3 secondary antibody (1:400, 711-167-003) was used, which is compatible with the use after of rabbit anti-ERG-Alexa647 (1:200).

### Flow cytometry and fluorescence-activated cell sorting

Mice organs were minced and digested with 2.5 mg/ml of Collagenase (Thermofisher) type I, 2.5 mg/ml Dispase II (Thermofisher) and 50 ng/ml of DNAseI (Roche) at 37 °C for 30 min to create a single cell suspension. Cells were filtered through a 70 μm filter to remove non-dissociated tissue. Erythroid cells were removed from cells suspensions with a Blood Lysis Buffer (0.15 M NH_4_Cl, 0.01 M KHCO_3_, and 0.01 M EDTA in distilled water) incubated for 10 min on ice. Cells suspensions were immediately analysed or blocked with in DPBS no Ca^2^+ or Mg^2^+, containing 3% Dialyzed FBS (Termofisher).

For endothelial cell analysis, cells were incubated at 4 °C for 30 min with APC conjugated rat anti-mouse CD31 (1:200, BD Pharmigen, 551262). DAPI was added prior to cell analysis. Anti-CD31-APC, MbTomato, or EYFP signals were gated with a negative control tissue.

Blood for FACS analysis (Fig. 3b, c and data supporting Supplementary Fig. 1) was collected by submandibular sampling and erythroid cells were also removed with the same blood lysis buffer described above. In the case of the LysM-Cre line analysis (Fig. 6a), blood cells were pre-incubated on ice with rat anti-Mouse CD16/CD32 (1:200, BD Pharmingen, 553141) and then immunostained for the different blood cell types using rat anti-CD11b-647 (1:200, BD Pharmingen, 557686), rat anti-CD45.2-APC-Cy7 (1:200, Tonbobio 25-0454), rat anti-Ter119-biotin (1:200, BD Biosciences, 553672), rat anti-CD3e-biotin (1:200, eBiosciences), rat anti-B220-biotin (1:200, BD Biosciences, 553085), and rat anti-Ly6G-PE-Cy7 (1:200, BD Biosciences, 560601). For the Vav1-Cre analysis, cells were incubated with APC rat anti-mouse CD45 (1:200, BD Biosciences, 561018).

For the isolation of cells from dissociated tissues (Fig. 3g; Fig. 4g–k; Fig. 5e; Fig. 7b, c), viable cells were selected by DAPI negative fluorescence. All viable cells were interrogated by examining FSC and SSC to select by size and complexity, and by comparing FSC-H and FSC-W repeated with SSC-H and SSC-W in order to discern single cells. An additional channel lacking any endogenous or fluorescent label was also acquired to detect and exclude autofluorescence. Cells were selected by their APC, endogenous EYFP, and endogenous MbTomato positive signal. Flow cytometry analysis and FACs were performed on Fortessa or Aria Cell Sorter or Synergy4L machines. Experiments were analyzed using DIVA software.

### Differentiation of bone marrow-derived macrophages

Bone marrow cells from LysM-Cre mice were flushed, passed through a 70 um filtered and plated 3 days in RMPI 1640 medium (supplemented with 10% FCS, Glut 2 mM, P/S, mouse 10 ng/uL M-CSF). Medium was renewed at Day 3. At Day 5, cells were dettached and sorted for YFP+ or MbTomato+ for downstream protein or RNA extraction and analysis. qRT-PCR was performed using specific primers for Epas1 (tgagttggctcatgagttgc and ttgctgatgttttccgacag) and controls 36b4 (agatgcagcagatccgcat and gttcttgcccatcagcacc) and cyclophilin (acaggtcctggcatcttgtc and catggcttccacaatgttca).

### Western blot analysis

For the analysis of protein expression, dissected organs were transferred to a reagent tube and frozen in liquid nitrogen. On the day of the immunoblotting the tissue was lysed with lysis buffer [(Tris-HCl pH = 8, 20 mM, EDTA 1 mM, DTT 1 mM, Triton X-100 1%, and NaCl 150 mM, containing protease inhibitors (P-8340 Sigma) and phosphatase inhibitors (Calbiochem 524629) and orthovanadate-Na 1 mM)] and homogenized with a cylindrical glass pestle. Sorted CD31-APC+ endothelial cells (Fig. 7b) or bone marrow-derived macrophages samples (Fig. 7c) were lysed with RIPA buffer (Sigma R0278) containing protease inhibitors (Sigma P-8340) and phosphatase inhibitors (Calbiochem 524629) and orthovanadate-Na 1 mM. Tissue/ cell debris was removed by centrifugation, and the supernatant was diluted in 3xSDS loading buffer and analysed by SDS–PAGE and immunoblotting. Membranes were blocked with BSA and incubated with primary antibodies diluted 1/1000 against Cre (Merck, 69050–3), Cdh5/VE-cadherin (BD Biosciences 555289) or β-Actin (Santa Cruz Biotechnologies, sc-47778).

### DNA or RNA profiling from FACS-sorted cells

Cells from different mice were sorted based on their YFP, Tomato, or anti-CD31-APC fluorescent signals in DPBS no Ca^2^+ or Mg^2^+, containing 3% Dialyzed FBS (Termofisher).

For DNA isolation, a minimum of 42,000 cells per sample, were sorted and spin down for 10 min at 350 g, and resuspended in 50 μl of lysis buffer prepared as follows: 25 ul of DirectPCR (Cell) Lysis Reagent for PCR (VIAGEN Cat #301-C) and 25 μl of distilled water and a final concentration of 0.4 mg/ml of proteinase K. Cells were incubated 55 °C overnight and then the proteinase was inactivated at 85 °C for 45 min. Semi-quantitative and competitive PCR was performed with 1 ul of DNA from the different samples using the primers mentioned in Supplementary Table 2. Groups of 3 or 4 primers were used per PCR reaction. Some of the PCRs served as control of DNA input, and the others corresponded to the different floxed genes sequences.

For RNA isolation, a minimum of 15,000 cells were sorted directly to RNeasy Mini Kit RLT buffer (Qiagen). RNA was extracted according to the Qiagen protocol. cDNA was synthetized with the High Capacity cDNA kit from Applied Biosystems (AB). cDNA was pre-amplified with the Taqman PreAmp Master Mix (AB) and after qRT-PCR with gene-specific Taqman assays (Cdh5: Mm00486938_m1; Notch1: Mm00435245_m1; KDR: Mm00440085_m1; Rbpj:Mm01217627_g1; GAPDH: Mm99999915_g1) and universal master mix was performed on a AB 7900 qRT-PCR machine.

### Image acquisition and analysis

ES cells, flat-mounted retinas, organs, and embryo sections with or without immunostaining were imaged at high resolution with a Zeiss LSM710 or Leica SP8/SP5 confocal microscopes^13^. ×10, ×20, or ×40 objectives were used for confocal scanning. Individual fields or tiles of large areas were acquired. Fiji/ImageJ was used to threshold, select and quantify objects in confocal micrographs. For charts in Fig. 3f, cell frequency was determined by counting the number of ERG + EC nuclei with MbTomato or EYFP or dual reporter expression (representative pictures Fig. 3d, e). ERG labels the nuclei of individual ECs, allowing their accurate quantification. In Fig. 3h and Supplementary Fig. 2a, up to six laser scanning confocal channels were acquired and quantified. ECs are very elongated and both MbTomato (546 nm) and EYFP (514 nm) proteins accumulate more in the cytoplasm around the nucleus (Fig. 3h, top right panel), being possible to count the total number of cells even in the absence of the incompatible nuclei ERG immunostaining channel. Besides this, we also counted on independent pictures the number of ERG+ nuclei/cells per MbTomato+ or YFP+ endothelial surface area, obtaining similar results. The fluorescent proteins with nuclei localization (H2B-Cherry (595 nm), H2B-EGFP (488 nm), and HA-H2B-Cerulean (anti-HA-647 antibody signal, 647 nm) were detected with separate and compatible laser excitation and detectors (Fig. 3h, bottom panels and Supplementary Fig. 2a). ImageJ was used to detect the number of nuclei (H2B-Cherry, H2B-EGFP or HA-H2B-Cerulean) objects in MbTomato+ or EYFP+ endothelial nuclei area (Fig. 3i and Supplementary Fig. 2a). ImageJ was also used to detect the total ERG+ (EC nuclei) objects in IsolectinB4 or MbTomato+ or MbYFP+ surface area in the pictures supporting the data in Fig. 4c–f. For the data shown in Fig. 5c, images representing large fields were selected and used to quantify the number of cells with detectable expression of MbTomato, Kdr, or Dll4 proteins. Quantification of PCR and Western gel bands intensity shown in Fig. 5e and Fig. 7 was done with Image J by calculating the mean grey value of each band and subtracting it from the background mean grey value. Indicated relative ratios in Fig. 5e represent the ratio between control and floxed PCR bands when comparing control samples (with no inducible genetic deletion) with MbTomato-negative and MbTomato-positive samples. In Fig. 7, fold change indicated represent the relative difference in Cre protein levels taking as a reference the protein loading controls (Cdh5 or B-actin).

### Statistical analysis

Two groups of samples with a Gaussian distribution were compared by unpaired two-tailed Student *t*-test. Comparisons among more than two groups were made by ANOVA followed by the Turkey pairwise comparison. Graphs represent mean ± SD as indicated in figure legends, and differences were considered significant at *p* < 0.05. All calculations were done in Excel and final datapoints analysed and represented with GraphPad Prism. No randomization or blinding was used, and animals or tissues were selected for analysis based on their genotype, the detected Cre-dependent recombination frequency, and quality of multiplex immunostaining. The sample size was chosen according to the observed statistical variation and published protocols.

### Reporting summary

Further information on research design is available in the Nature Research Reporting Summary linked to this article.

## Supplementary information

Supplementary Information

Reporting Summary

Source Data

## Data Availability

A previously constructed plasmid Addgene plasmid #8395 was used in this work. All data supporting the findings of this study are available from the corresponding author upon request. This includes raw data such as unprocessed original pictures and independent replicates, which are not displayed in the manuscript, but are included in the data analysis in the form of graphs. The source data underlying all figures numeric or chart data are provided as a Source Data file.
