# Supplementary material for: iSuRe-Cre is a genetic tool to reliably induce and report Cre-dependent genetic modifications
Source: Nat Commun. 2019 May 22;10:2262. doi: 10.1038/s41467-019-10239-4 (PMC6531465; doi:10.1038/s41467-019-10239-4)
Supplement: Supplementary file 1 — Supplementary Information [file 41467_2019_10239_MOESM1_ESM.pdf]

Supplementary Information

iSuRe-Cre is a new genetic tool to reliably induce and report Cre-dependent genetic modifications

Macarena Fernández-Chacón, Verónica Casquero-García, Wen Luo, Federica Francesca Lunella, Susana Ferreira Rocha, Sergio Del Olmo-Cabrera, Rui Benedito.

It includes;
Supplementary Figures 1-4 and Supplementary Tables 1 and 2.

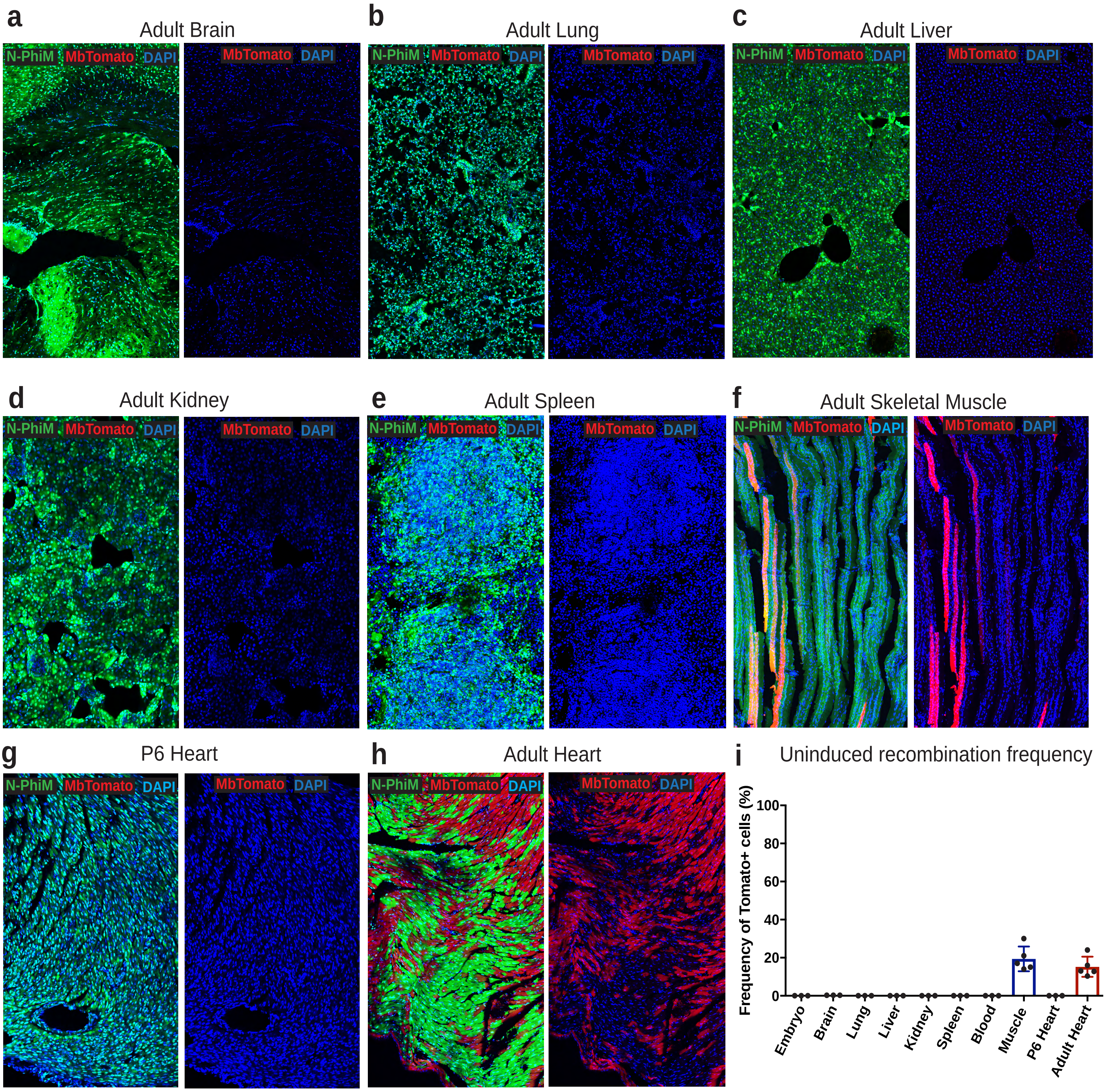

j

Compound floxed animals containing the Tg-iSuRe-Cre allele, born at mendelian ratios and healthy	Dll4/Kdr $^{flox/flox}$	n= 17
	Fgfr1/Fgfr2/Kdr/Rbpj $^{flox/flox}$	n= 24
	Rbpj/Myc/Mycn $^{flox/flox}$	n= 35
	Hif1/Hif2 $^{flox/flox}$	n= 21

Supplementary Figure 1. The Tg(iSuRe-Cre) is expressed in all organs and self-recombines in some adult myocytes.
a-e) Representative confocal micrographs of different mouse organs, showing that the Tg(iSuRe-Cre) allele is expressed in most cell types (N-PhiM-positive) and does not self-recombine (MbTomato-negative) in the cells of the indicated organs.
f) A fraction of adult skeletal muscle fibres self-recombine the Tg(iSuRe-Cre) allele and are MbTomato+.
g, h) A fraction of adult, but not postnatal (P6), cardiomyocytes also self-recombine the allele (MbTomato+).
i) Frequency of non-induced recombination in the embryo, the postnatal heart, and the indicated adult organs. MbTomato+ cells were counted on several immunostained sections and also by whole-organ FACS analysis. Self-recombination of the Tg(iSuRe-Cre) allele is found in only a fraction of quiescent skeletal muscle and cardiac myocytes.
j) Number and genotype of animals obtained with compound floxed alleles and the Tg(iSuRe-Cre) allele.
Error bars indicate StDev; each dot represents the mean result obtained per animal and organ.

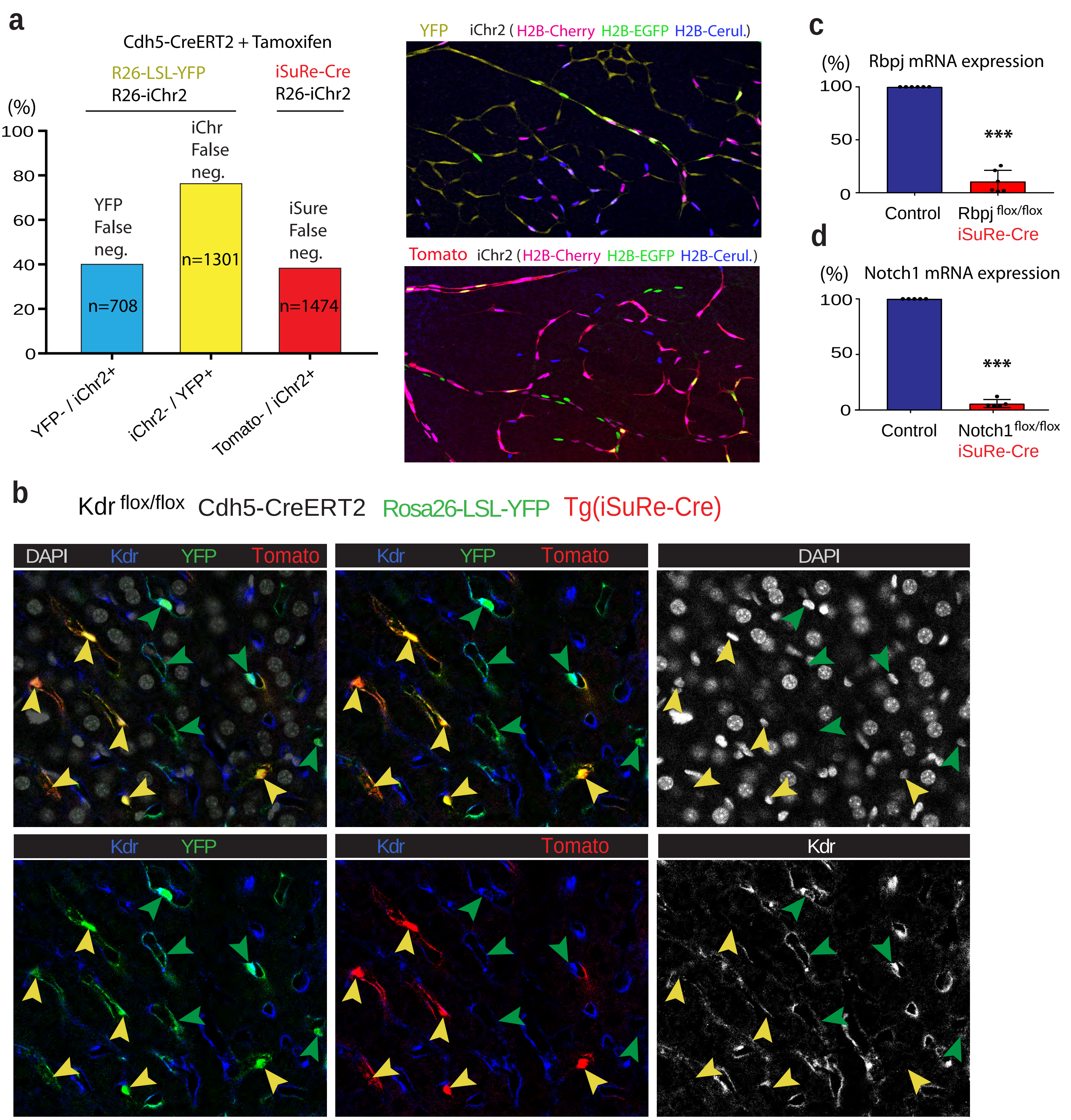

Supplementary Figure 2. Comparison of conventional and the Tg(iSuRe-Cre) reporter alleles.
a) Chart showing the % of cells with the indicated combination of reporters expression, obtained in animals with the indicated genotypes (see figures on the right). The numbers obtained are an indication of relative reporter false-negatives (cells not expressing a given reporter (-), but expressing another reporter). The Rosa26-iChr2 reporter gives more false-negatives, indicating a relatively lower recombination efficiency.
b) 4-channel confocal micrographs showing Kdr immunostaining signals in YFP+ and Tomato+ cells of animals with the indicated genotype. DAPI staining was used to identify the nuclei of ECs and segment objects during quantification. iSuRe-Cre Tomato+ cells (yellow arrowheads) recombined the Rosa26-LSL-YFP+ allele, express YFP, and deleted the gene Kdr. In contrast, a significant fraction of YFP+ cells (green arrowheads), still express Kdr. Note that ECs have a very elongated shape, and mutant (Kdr-) cells may be surrounded by wildtype (Kdr+) cells. Only the stronger Kdr signals in the cell body (overlapping with DAPI+ nuclei) was used for the quantifications. Quantification data is shown in Fig. 4g bottom chart.
c, d) Charts showing the relative Rbpj and Notch1 mRNA levels in iSure-Cre Tomato + cells (cells were isolated from n=6 and n=5 animals per group respectively).
Error bars in c and d indicate StDev; *** p< 0.0005. Two-tailed unpaired T-test. Source data are provided as a Source Data file.

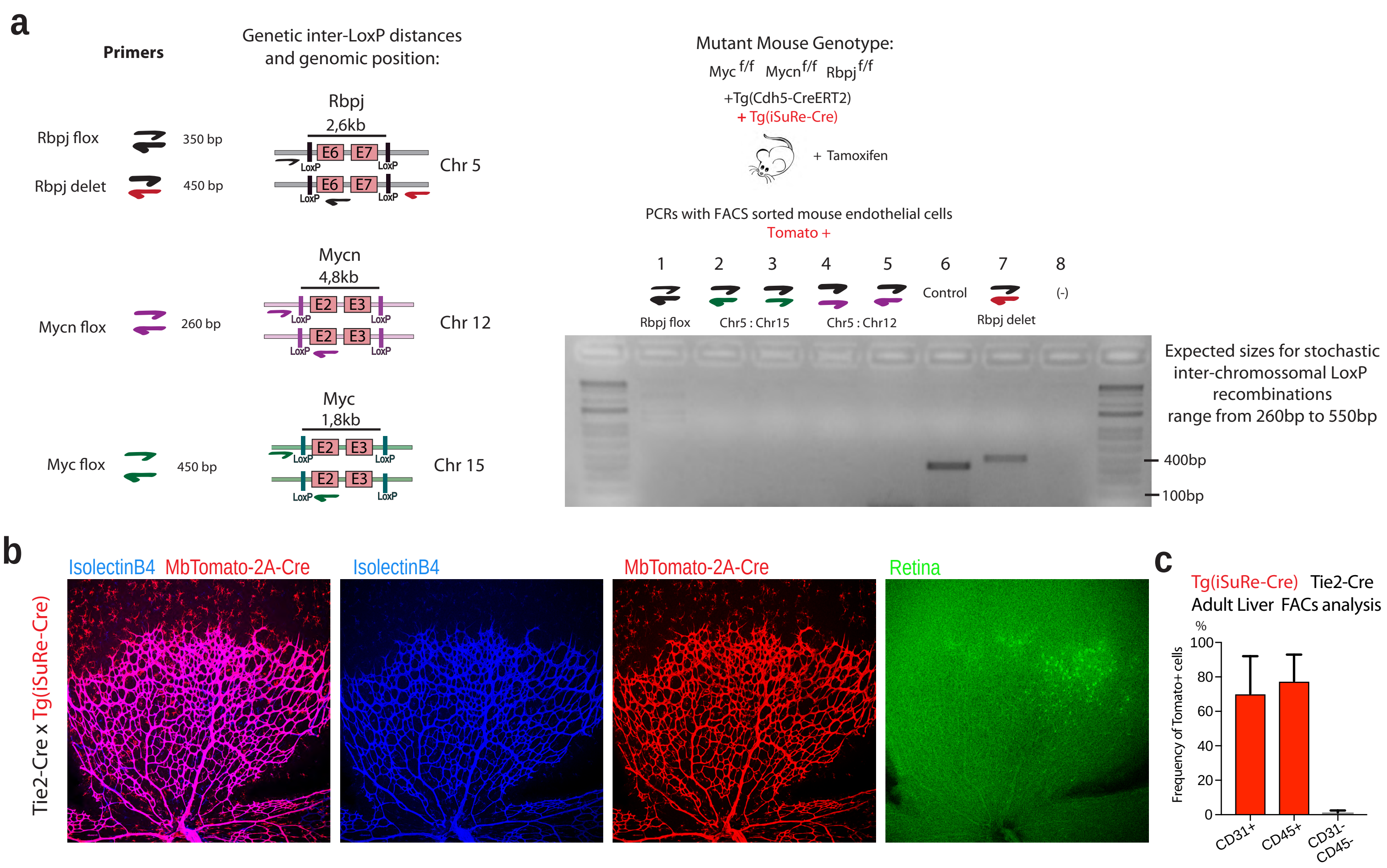

Supplementary Figure 3. Specificity and interchromosomal recombination analysis in Tg(SuRe-Cre) mice.
a) Schematic representation of the primers used to detect recombination among non-homologous chromosomes containing floxed genes. In cells having 6 alleles floxed and expressing MbTomato-2A-Cre, we could only detect the predicted Rbpj genetic deletion, and not the floxed allele or the non-homologous interchromosomal recombination.
b) Representative confocal micrographs of retinas showing that in animals with the Tie2-Cre and Tg(iSuRe-Cre) alleles, MbTomato is only expressed in endothelial (isolectinB4) and blood cells (macrophages, red), and not in the many other cell types present in the retinal tissue (green channel).
c) Chart showing that among all liver cells, only CD31+ endothelial and CD45+ blood cells express the MbTomato reporter in animals with the indicated genotype (n=4).
Error bars indicate StDev. Source data are provided as a Source Data file.

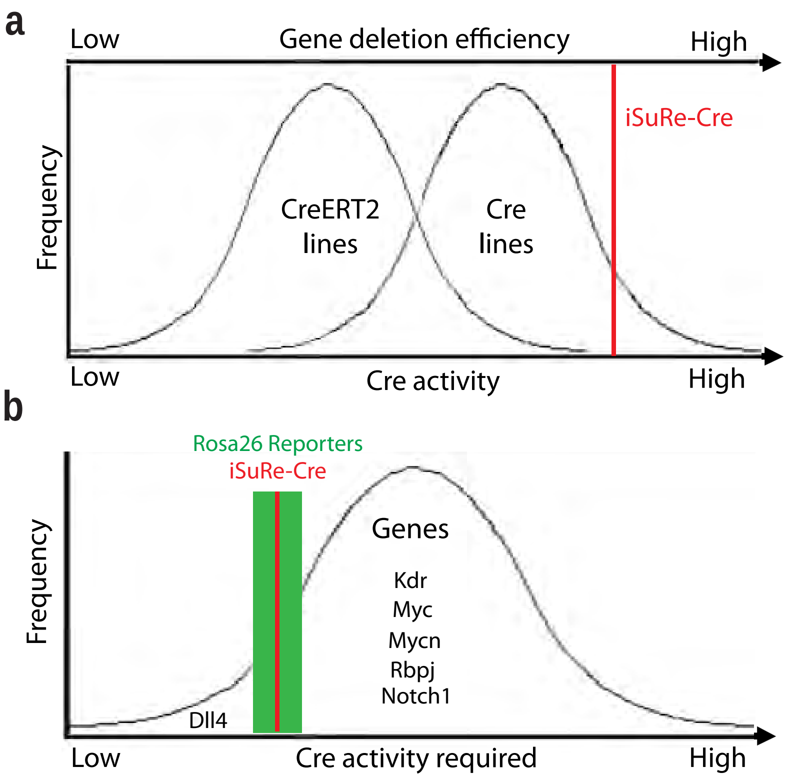

c

	Advantages	Disadvantages
**Tissue-specific Cre**<br>Ex: Tie2-Cre	- Expression of constitutively active Cre Protein.<br>- Higher recombination rates.	- Maybe not completely penetrant (variable).<br>- Promoter may be weakly expressed in many cells.<br>- Not inducible - no temporal control of genetic modification.
**Tissue-specific CreERT2**<br>Ex: Cdh5-CreERT2	- Inducible by tamoxifen. Temporal control of genetic deletion.	- CreERT2 protein needs to be induced by tamoxifen.<br>- High doses of tamoxifen may induce cellular toxicity.<br>- Recombination activity is significantly lower than Cre.<br>- Variable inducibility rates.
**Reporters of Cre**<br>Ex: Rosa26-LSL-EYFP	- Inducible by any Cre or CreERT2 expressing allele.<br>- Label cells with weak or strong Cre activity.	- Reporter expression does not correlate with gene deletion.<br>- May under or over-report intended gene deletion.
**Dual Reporter-Cre**<br>**Tg(iSuRe-Cre)**	- Inducible by any Cre or CreERT2 expressing allele.<br>- Stronger and equimolar expression of both Reporter and Cre.<br>- Very high correlation of reporter expression with gene deletion.<br>- Enables the efficient induction of multiple genetic deletions in single cells or entire tissues, particularly with CreERT2 lines.	- Leaky and self-recombines in a fraction of adult myocytes.<br>- High Cre activity in reporter expressing cells may need control

Supplementary Figure 4. Correlation between Cre activity and gene deletion.
a) Chart representing the theoretically predicted and experimentally observed correlation between Cre activity and efficiency of gene deletion. Constitutive Cre-expressing lines achieve, in general, higher gene deletion efficiencies than tamoxifen-inducible CreERT2 lines. Given the higher Cre expression provided by the iSuRe-Cre allele, the observed gene deletion efficiency is higher with this line than with most other CreERT2 or Cre lines.
b) Chart representing the theoretically predicted and experimentally observed correlation between Cre activity and the recombination of reporter alleles or full deletion of genes. Like other conventional Rosa26-reporter alleles, the iSuRe-Cre allele is efficiently induced/recombined at relatively low levels of Cre activity. With the exception of the gene Dll4, all the other experimentally tested genes (Kdr, Myc, Mycn, Rbpj and Notch1) are more difficult to delete/recombine than the iSuRe-Cre or Rosa26-reporter alleles. The easier induction of iSuRe-Cre recombination is followed by robust gene deletion because cells express higher levels of both the reporter and Cre.
c) Table highlighting the main advantages and disadvantages of each type of Cre or reporter mouse lines.

		CONTROL Littermates						Tg(iSuRe-Cre)						
		Control #1	Control #2	Control #3	Control #4	Control #5	**Mean ± SD**	Mutant #1	Mutant #2	Mutant #3	Mutant #4	Mutant #5	**Mean ± SD**	**p-value**
**M-MODE SAX**	**IVS;d**	0.643	0.796	0.735	0.766	0.735	**0.735 ± 0.057**	0.643	0.674	0.682	0.674	0.735	**0.681 ± 0.033**	**0.109**
	**LVID;d**	4.594	4.196	4.594	3.920	4.716	**4.403 ± 0.334**	4.410	4.134	4.134	4.196	4.318	**4.238 ± 0.122**	**0.329**
	**LVID;s**	3.430	3.277	3.675	2.664	3.675	**3.344 ± 0.416**	3.522	3.093	2.971	3.124	3.399	**3.221 ± 0.230**	**0.580**
	**LVPW;d**	0.735	0.704	0.674	0.674	0.704	**0.698 ± 0.026**	0.796	0.674	0.613	0.766	0.735	**0.716 ± 0.074**	**0.613**
	**%EF**	50.063	44.648	41.057	60.815	44.577	**48.232 ± 7.736**	41.427	50.186	54.867	50.721	43.483	**48.137 ± 5.542**	**0.983**
	**%FS**	25.333	21.898	20.000	32.031	22.078	**24.268 ± 4.744**	20.139	25.185	28.148	25.547	21.277	**24.059 ± 3.291**	**0.938**
	**LV Mass**	122.187	116.852	125.655	98.667	135.146	**119.701 ± 13.516**	120.433	99.052	84.918	110.642	119.410	**106.890 ± 14.987**	**0.194**
	**LV Mass C**	97.750	93.482	100.524	78.933	108.117	**95.761 ± 10.813**	96.346	79.242	67.934	88.513	95.528	**85.512 ± 11.990**	**0.194**
	**LV Vol;d**	97.026	78.385	97.026	66.717	103.190	**88.469 ± 15.313**	88.159	75.705	75.705	78.385	83.895	**80.369 ± 5.490**	**0.298**
	**LV Vol;s**	48.452	43.388	57.190	26.143	57.190	**46.472 ± 12.811**	51.637	37.711	34.168	38.627	47.415	**41.911 ± 7.302**	**0.509**
**MITRAL FlOW**	**E**	628.017	853.460	898.279	810.107	798.078	**797.588 ± 102.679**	523.348	990.577	909.267	873.588	810.108	**821.377 ± 178.905**	**0.803**
	**A**	257.648	430.756	358.427	376.981	481.253	**381.012 ± 84.065**	110.708	316.824	307.241	378.421	332.866	**289.212 ± 103.459**	**0.162**
	**E/A**	2.438	1.981	2.506	2.149	1.658	**2.146 ± 0.346**	4.727	3.127	2.959	2.309	1.720	**2.968 ± 1.130**	**0.159**
	**IVRT**	16.667	14.167	18.333	15.000	19.167	**16.666 ± 2.125**	23.000	15.000	14.167	16.667	17.500	**17.266 ± 3.465**	**0.750**
	**HR**	397.000	457.000	382.000	453.000	445.000	**426.800 ± 34.730**	386.000	421.000	429.000	421.000	484.000	**428.200 ± 35.351**	**0.951**

Primer Name	Primer Sequence	PCR Ta	Band Size	Purpose
Rosa26 GT Accu F	TCAGAGAGCCTCGGCTAGGTAGGG	65C	**1,200bp**	To detect gene targeting in Rosa26 locus
INS GT Accu R	ACTCCAGGACGGAGTCAGTGAGGA			
MTomato F	CCGCTACCTGGTGGAGTTCA	60C	**350bp**	To detect Tg(iSuRe-Cre) allele
Cre R	TCCCTGAACATGTCCATCAG			
Control Dll4 Wt F	GTGCTGGGACTGTAGCCACT	60C	**430bp**	To have an internal PCR control for the Mycn, Rbpj and Notch1 floxed PCRs
Control Dll4 Wt R	TGTTAGGGATGTCGCTCTCC			
Myc floxed F	TTTTCTTTCCGATTGCTGAC	55C	**450bp**	To detect the Myc floxed allele
Myc floxed R	TAAGAAGTTGCTATTTTGGC			
Mycn floxed F	GTCGCGCTAGTAAGAGCTGAGATC	60C	**260bp**	To detect the Mycn floxed allele
Mycn floxed R	CACAGCTCTGGAAGGTGGGAGAAAGTTGAAGCGTCTCC			
Rbpj floxed F	ATAATTTGCCAAGCCAAAGC	60C	**350bp**	To detect the Rbpj floxed allele
Rbpj floxed R	GCTCCCCACTGTTGTGAACT			
Control Jag1 Wt F	TGAACTCAGGACAGTGCTCT	55C	**389bp**	To have an internal PCR control for the Myc floxed PCR
Control Jag1 Wt R	GTTTCAGTGTCTGCCATTGC			
Sv40 pA F<br>MTomato R<br>Chr17 F<br>Chr17 R	CCCCCTGAACCTGAAACATA<br>CCTTGCTCACCATGGTCTTG<br>GCTCTGCATGTTGCAAGAAA<br>GTGTCTGTACCAGGTTGGTTTG	60C	**260bp Mutant band**<br>**104bp Wt band**	To detect Tg(iSuRe-Cre) allele and distinguish heterozygous from homozygous
Notch1 floxed F	CTG ACT TAG TAG GGG GAA AAC	60C	**500bp**	To detect the Notch1 floxed allele
Notch1 floxed R	AGT GGT CCA GGG TGT GAG TGT			
