# Supplementary material for: iSuRe-Cre is a genetic tool to reliably induce and report Cre-dependent genetic modifications
Source: Nat Commun. 2019 May 22;10:2262. doi: 10.1038/s41467-019-10239-4 (PMC6531465; doi:10.1038/s41467-019-10239-4)
Supplement: Supplementary file 2 — Reporting Summary [file 41467_2019_10239_MOESM2_ESM.pdf]

nature research

Corresponding author(s): *Double-blind peer review submissions: write DBPR and your manuscript number here instead of author names.*

Last updated by author(s): *YYYY-MM-DD*

Reporting Summary

Nature Research wishes to improve the reproducibility of the work that we publish. This form provides structure for consistency and transparency in reporting. For further information on Nature Research policies, see Authors & Referees and the Editorial Policy Checklist.

Statistics

For all statistical analyses, confirm that the following items are present in the figure legend, table legend, main text, or Methods section.

n/a	Confirmed	
☒	☐	The exact sample size (*n*) for each experimental group/condition, given as a discrete number and unit of measurement
☒	☐	A statement on whether measurements were taken from distinct samples or whether the same sample was measured repeatedly
☒	☐	The statistical test(s) used AND whether they are one- or two-sided<br>*Only common tests should be described solely by name; describe more complex techniques in the Methods section.*
☐	☐	A description of all covariates tested
☒	☐	A description of any assumptions or corrections, such as tests of normality and adjustment for multiple comparisons
☒	☐	A full description of the statistical parameters including central tendency (e.g. means) or other basic estimates (e.g. regression coefficient) AND variation (e.g. standard deviation) or associated estimates of uncertainty (e.g. confidence intervals)
☐	☐	For null hypothesis testing, the test statistic (e.g. *F*, *t*, *r*) with confidence intervals, effect sizes, degrees of freedom and *P* value noted<br>*Give P values as exact values whenever suitable.*
☐	☐	For Bayesian analysis, information on the choice of priors and Markov chain Monte Carlo settings
☐	☐	For hierarchical and complex designs, identification of the appropriate level for tests and full reporting of outcomes
☐	☐	Estimates of effect sizes (e.g. Cohen's *d*, Pearson's *r*), indicating how they were calculated

Our web collection on statistics for biologists contains articles on many of the points above.

Software and code

Policy information about availability of computer code

Data collection	Data was collected with the indicated Confocal Microscopes, FACS analysers/sorters, and qRT-PCR machines software.
Data analysis	Numerical data was analysed with Graphpad. Microscope images were analysed with ImageJ/FIJI.

For manuscripts utilizing custom algorithms or software that are central to the research but not yet described in published literature, software must be made available to editors/reviewers. We strongly encourage code deposition in a community repository (e.g. GitHub). See the Nature Research guidelines for submitting code & software for further information.

Data

Policy information about availability of data

All manuscripts must include a data availability statement. This statement should provide the following information, where applicable:

- Accession codes, unique identifiers, or web links for publicly available datasets
- A list of figures that have associated raw data
- A description of any restrictions on data availability

All data supporting the findings of this study are available from the corresponding author upon request. This includes raw data such as unprocessed original pictures and independent replicates, which are not directly displayed in the manuscript, although included in the data analysis in the form of graphs.

Field-specific reporting

Please select the one below that is the best fit for your research. If you are not sure, read the appropriate sections before making your selection.

☒ Life sciences ☐ Behavioural & social sciences ☐ Ecological, evolutionary & environmental sciences

For a reference copy of the document with all sections, see nature.com/documents/nr-reporting-summary-flat.pdf

Life sciences study design

All studies must disclose on these points even when the disclosure is negative.

Sample size	The sample size was chosen according to the observed statistical variation and published protocols. Sample size was determined taking into account the expected experimental variability or a posteriori based on the statistical analysis of the data. Two groups of samples with a Gaussian distribution were compared by an unpaired two-tailed Student t-test. For comparisons among more than two groups were made by ANOVA followed by the Turkey pairwise comparison for Gaussian distributed groups. Graphs represent mean +/- SD or SEM as indicated, and differences were considered significant at $p < 0.05$.
Data exclusions	Data was excluded only if technical problems were detected. These include unexpected animal behaviour or morbidity after tamoxifen or tumor injections, insufficient gene deletion or technical problems detected with immunostaining or microscopy analysis.
Replication	As it can be seen in the submitted article, we repeated the experiments several times to guarantee maximum reproducibility. In addition, the main conclusions are supported by different experiments using different mouse models or cell lines.
Randomization	No randomization was used, and animals/tissues were selected for analysis based on their genotype, the detected Cre-dependent recombination frequency, and quality of multiplex immunostaining.
Blinding	In the majority of cases, investigators were not blinded during data collection or analysis. ImageJ/FIJI software was used to analyse the microscopy data in an automatic and objective manner.

Reporting for specific materials, systems and methods

We require information from authors about some types of materials, experimental systems and methods used in many studies. Here, indicate whether each material, system or method listed is relevant to your study. If you are not sure if a list item applies to your research, read the appropriate section before selecting a response.

Materials & experimental systems

n/a	Involved in the study	
☐	☒	Antibodies
☐	☒	Eukaryotic cell lines
☒	☐	Palaeontology
☐	☒	Animals and other organisms
☒	☐	Human research participants
☒	☐	Clinical data

Methods

n/a	Involved in the study	
☒	☐	ChIP-seq
☐	☒	Flow cytometry
☒	☐	MRI-based neuroimaging

Antibodies

Antibodies used	We include all this info in the Methods/Immunohistochemistry section. Primary antibody (1:400, Rabbit Anti-PhiYFP, AB602, Evrogen) The following primary antibodies were used: goat anti-Dll4 (1:200, R&D systems, AF1389); rat anti-VEGFR2 (1:200, BD Pharmingen, 550549), rabbit anti-Dsred (1:200, Clontech, 632496) and rabbit anti-ERG-Alexa647 (1:200, Abcam, Ab110639). To detect MbTomato in the same section as ERG, endogenous signals were scanned or rabbit anti-Dsred plus a Fab fragment CY3 secondary antibody (1:400, 711-167-003) was used, which is compatible with the use after of rabbit anti-ERG-Alexa647 (1:200). primary antibodies diluted 1/1000 against Cre (Merck, 69050-3), Cdh5/VE-cadherin (BD Biosciences 555289) or β-Actin (Santa Cruz Biotechnologies, sc-47778).
Validation	Commercially available and publication validated antibodies.

Eukaryotic cell lines

Policy information about cell lines

Cell line source(s)	We used published mouse ES cells (G4) from Andreas Nagy laboratory.
Authentication	Cells lines were not formally authenticated, but they had the reported phenotype and biology.
Mycoplasma contamination	negative
Commonly misidentified lines (See ICLAC register)	No commonly misidentified cell lines were used.

Animals and other organisms

Policy information about studies involving animals; ARRIVE guidelines recommended for reporting animal research

Laboratory animals	We used laboratory mice (mus musculus) with C57Bl6 or C57Bl6x129Sv genetic backgrounds. We used both male and female mice and we do not expect our data to be influenced by animal sex. Details about the transgenic or gene-targeted allleles used are provided in the paper text.
Wild animals	*Provide details on animals observed in or captured in the field; report species, sex and age where possible. Describe how animals were caught and transported and what happened to captive animals after the study (if killed, explain why and describe method; if released, say where and when) OR state that the study did not involve wild animals.*
Field-collected samples	*For laboratory work with field-collected samples, describe all relevant parameters such as housing, maintenance, temperature, photoperiod and end-of-experiment protocol OR state that the study did not involve samples collected from the field.*
Ethics oversight	As indicated in the manuscript, all experiments were conducted according to local authorities and following standard procedures and regulatory requirements.

Note that full information on the approval of the study protocol must also be provided in the manuscript.

Flow Cytometry

Plots

Confirm that:

- [ ] The axis labels state the marker and fluorochrome used (e.g. CD4-FITC).
- [ ] The axis scales are clearly visible. Include numbers along axes only for bottom left plot of group (a 'group' is an analysis of identical markers).
- [ ] All plots are contour plots with outliers or pseudocolor plots.
- [ ] A numerical value for number of cells or percentage (with statistics) is provided.

Methodology

Sample preparation	*Describe the sample preparation, detailing the biological source of the cells and any tissue processing steps used.*
Instrument	*Identify the instrument used for data collection, specifying make and model number.*
Software	*Describe the software used to collect and analyze the flow cytometry data. For custom code that has been deposited into a community repository, provide accession details.*
Cell population abundance	*Describe the abundance of the relevant cell populations within post-sort fractions, providing details on the purity of the samples and how it was determined.*
Gating strategy	*Describe the gating strategy used for all relevant experiments, specifying the preliminary FSC/SSC gates of the starting cell population, indicating where boundaries between "positive" and "negative" staining cell populations are defined.*

- [ ] Tick this box to confirm that a figure exemplifying the gating strategy is provided in the Supplementary Information.
